# An Adaptive Bounded Hybrid A* Planner for Autonomous Mining Trucks in Confined Unstructured Mining Environments

**DOI:** 10.3390/s26185720

**Published:** 2026-09-09

**Authors:** Zijie Meng, Ruixin Zhang

**Affiliations:** School of Energy and Mining Engineering, China University of Mining & Technology-Beijing, Beijing 100083, China

**Keywords:** autonomous mining trucks, adaptive expansion, Hybrid A*, bounded continued search

## Abstract

Automated valet-parking trajectory planning has been widely studied in structured environments, whereas its counterpart in unstructured environments remains insufficiently explored. The working face of an open-pit mine is a typical unstructured production environment, where autonomous mining trucks (AMTs) must perform collision-free and kinematically feasible maneuvers under irregular boundaries, locally confined passages, large vehicle footprints, and strict terminal pose requirements. To address this problem, we introduce an adaptive bounded Hybrid A* planner for AMT maneuver planning at mining working faces. The proposed planner integrates local-geometry-aware top-K motion primitive scheduling, adaptive rollout-length assignment, progress-reward-based dual-cost correction, and bounded continuation after the first feasible Reeds–Shepp closure. By ranking and filtering motion primitives according to obstacle clearance, maneuver margin, goal distance, and heading alignment, the planner reduces ineffective successor generation during search. By adapting rollout lengths to local spatial constraints and primitive curvature, it improves the consistency between expansion scale and environmental structure. By retaining the first feasible Reeds–Shepp connection as an initial upper bound and continuing bounded refinement, it improves the final returned path beyond the first feasible solution. Comparative studies demonstrate that, relative to the conventional Hybrid A* baseline, the proposed planner reduces the average final planning time, generated nodes, and final path cost by 54.73%, 74.10%, and 12.76%, respectively, in the abstract mine-like scenarios. In the real-map-derived mining working-face scenarios, the corresponding reductions are 25.12%, 37.98%, and 7.15%, respectively. These results indicate that the proposed method reduces search redundancy while improving the efficiency–quality trade-off in confined and geometrically irregular working-face environments.

## 1. Introduction

**Figure 1:**
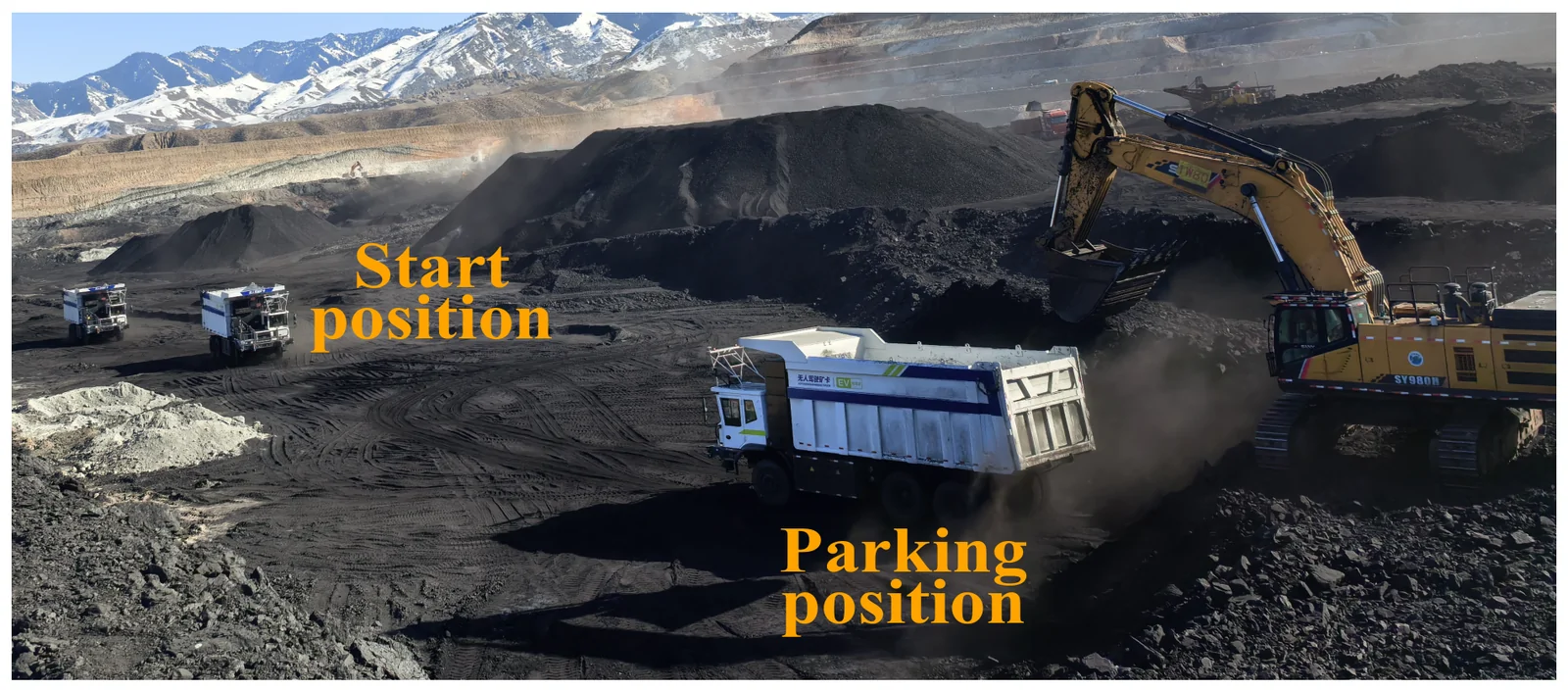
Typical unstructured scenario of the open-pit mine working face.

Automated valet-parking trajectory planning has been extensively studied in structured environments, where parking slots, driving boundaries, and terminal poses are usually well defined [1,2]. In contrast, trajectory planning in unstructured environments usually lacks reliable reference lines, encounters irregular obstacles, and requires mixed forward/backward maneuvers [3]. The working face of an open-pit mine is a typical unstructured production environment [4,5,6]. In this area, autonomous mining trucks (AMTs) must complete loading, unloading, docking, queuing, and short-distance maneuvering tasks under irregular boundaries, large vehicle footprints, wide turning radii, and strict terminal pose requirements, as depicted in Figure 1.

Search-based planners with motion primitives are widely used for low-speed nonholonomic maneuver planning. Hybrid A* is a representative method because it combines graph search with continuous vehicle-state propagation and can generate kinematically feasible paths for car-like systems [7]. Reeds–Shepp (RS) curves are often used as analytical connections to accelerate terminal pose reaching [8]. These properties make Hybrid A* a suitable baseline for AMT path planning at open-pit mine working faces.

Recent studies have improved narrow-space motion planning through intermediate-point-guided Hybrid A* search, richer terminal-set or target-tree construction, multi-resolution motion-primitive sampling, and optimization-based path refinement [9,10,11]. These methods can reduce search effort, enlarge terminal reachability, or improve path quality in constrained parking environments. However, most of them are developed for passenger-vehicle-scale parking tasks and either rely on structured parking geometry, externally generated guidance, or subsequent numerical optimization. Mining-oriented studies have considered loading/dumping and other operation-specific trajectory-planning tasks [12,13], but the coexistence of large vehicle footprints, irregular working-face boundaries, local bottlenecks, heterogeneous free-space scales, and mixed forward–reverse maneuvering remains insufficiently addressed. This gap motivates an adaptive search strategy that directly modifies the expansion process of Hybrid A* according to local working-face geometry.

However, directly applying conventional Hybrid A* to AMT working face scenarios still has three limitations. First, fixed rollout lengths cannot adapt to the coexistence of open regions and locally narrow passages. Short rollouts cause redundant expansion in open areas, whereas long rollouts may lead to collision or expansion deadlock in confined spaces. Second, exhaustive primitive expansion evaluates many low-value candidates at each node, increasing collision-checking cost for AMT with large swept areas. Third, early termination after the first feasible RS closure may return a feasible but high-cost path with unnecessary reversing, gear switching, or steering correction.

To address these limitations, this paper proposes an adaptive bounded Hybrid A* planner for AMT path planning at open-pit mine working faces. The proposed planner uses local geometric descriptors, including obstacle clearance, directional maneuver margin, goal distance, and heading alignment, to rank and filter a fixed primitive pool through top-K motion primitive scheduling. It further assigns adaptive rollout lengths according to local obstacle proximity, maneuver margin, and primitive curvature. A progress-reward-based dual-cost correction is introduced to reduce the cost-comparison bias caused by variable-length rollout. Finally, the first feasible RS-connected path is treated as an initial upper-bound candidate, and bounded continuation is performed to search for lower-cost feasible paths.

The main contributions of this paper are summarized as follows:

- A local-geometry-aware top-K motion primitive scheduling strategy is proposed to reduce ineffective successor generation in confined and irregular AMT working face scenarios.
- An adaptive rollout-length assignment mechanism is developed to match the expansion scale with local spatial conditions and primitive curvature.
- A progress-reward-based dual-cost correction is introduced to separate true path evaluation from search ordering under variable-length rollout.
- A bounded continuation strategy after the first feasible RS closure is proposed to refine the final returned path beyond the first feasible solution.

Comparative experiments in abstract mine-like scenarios and real-map-derived mining working-face scenarios validate the effectiveness of the proposed planner in reducing search redundancy, improving computational efficiency, and enhancing final path quality.

The remainder of this paper is organized as follows. Section 2 reviews related work. Section 3 formulates the AMT path planning problem. Section 4 presents the proposed planner. Section 5 describes the experimental setup and discusses the experimental results. Section 6 concludes the paper.

## 2. Related Work

Trajectory planning methods for low-speed maneuvering in unstructured environments can be broadly divided into geometry-based planners, sampling-based planners, optimization-based planners, and learning-based planners [14,15,16]. Geometry-based planners generate paths using predefined geometric primitives according to terminal pose, vehicle size, and minimum turning radius. They are computationally efficient and effective in standardized parallel parking, reverse parking, and angle parking scenarios [9,17,18]. However, their applicability is limited in open-pit mine working faces because the boundaries, obstacles, and terminal approach regions are usually irregular rather than standardized. Optimization-based motion-planning and control methods formulate kinematic or dynamic behavior, actuation limits, positioning or terminal requirements, and collision-avoidance constraints within optimal-control or numerical-optimization frameworks [19,20,21,22]. These methods can handle precise obstacle constraints, but the resulting problems are often nonlinear and non-convex, especially when irregular obstacles and large vehicle footprints are considered. Learning-based planners have also been used to generate parking or maneuvering trajectories through supervised learning, reinforcement learning, or hybrid learning-control frameworks [23,24,25]. Although learning-based methods can provide stable inference time, their generalization ability in narrow, cluttered, and safety-critical unstructured environments still depends heavily on the diversity and fidelity of training scenarios.

Sampling-based planners are more directly related to this work. They can be divided into control-space sampling and state-space sampling methods. Control-space sampling methods, such as Hybrid A*, expand a trajectory tree by rolling out fixed motion primitives under vehicle kinematic constraints [7]. Deterministic primitive sampling can also pre-generate a connectivity graph under a given spatial and angular resolution, after which a feasible path is obtained by graph search [26]. These methods naturally incorporate nonholonomic constraints and are suitable for AMT, whose large dimensions, wide turning radii, and restricted reverse maneuvers must be explicitly considered. However, conventional control-space sampling does not directly incorporate environmental information into the primitive sampling process. Its efficiency and success rate are strongly tied to the primitive resolution: dense primitives improve feasibility but increase solution time, whereas sparse primitives reduce computation but may miss feasible maneuvers in narrow passages [27,28]. State-space sampling methods, including RRT-based variants, can use environmental information more flexibly and can be probabilistically complete [29,30,31,32,33]. Nevertheless, narrow spaces remain a bottleneck because random samples have a low probability of connecting to the existing trajectory tree without collision.

Several studies have attempted to improve trajectory planning in narrow or unstructured spaces. For standardized parking tasks, early geometry-based methods focused on retrieving feasible parallel-parking paths in extremely limited parking spaces by exploiting predefined path patterns, minimum turning-radius constraints, and terminal-pose geometry [34,35]. Although these methods are efficient, their reliance on standardized parking geometry limits their applicability when obstacle shapes and free-space boundaries become irregular. To improve applicability in narrow environments, optimization-based path planning methods have been proposed to adjust path shapes under vehicle kinematic and terminal constraints [36]. More recent studies further improve terminal reliability by transforming the parking target or enlarging the reachable terminal set. For example, mirroring-based parking target construction converts bidirectional parking into a more reliable planning problem and improves completion efficiency in constrained parking scenarios [37]. Target-set and target-tree methods also replace a single terminal pose with a set of backward parking paths or continuous-curvature target branches, allowing sampling-based planners to connect the initial state to a richer terminal structure [10,38]. These methods improve narrow-space reachability, but they are still mainly designed for passenger-car parking rather than AMT maneuver planning at open-pit mine working faces.

In addition to target-structure design, researchers have improved the front-end search process itself. Multi-resolution motion-primitive sampling has been introduced to alleviate redundant expansion in non-convex feasible spaces, allowing the planner to discover farther and more valuable nodes within a single expansion process [11]. Replanning and stitching strategies have also been studied to handle sudden environmental changes during automated parking, where an updated local trajectory is generated by connecting the current vehicle state to the remaining planned trajectory [16]. Learning-based methods provide another direction. Graph neural networks, reinforcement learning, and imitation learning have been used to generate initial trajectories, hybrid policies, or end-to-end parking paths [39,40,41,42]. These methods can reduce online computation or stabilize inference time, but their robustness in narrow and cluttered environments depends strongly on the diversity of training data and the coverage of difficult homotopy classes. For mining-related applications, trajectory planning for autonomous trucks has been studied in open-pit mine transport sections, loading/dumping scenarios, and mining-site loading tasks [12,13,43]. These studies confirm the importance of vehicle kinematics, terminal-pose accuracy, and operation-specific maneuver quality. However, existing methods still do not sufficiently address AMT path planning at the open-pit mine working face, where irregular boundaries, local bottlenecks, mixed forward/backward maneuvers, and large swept areas coexist.

The above studies reveal three limitations that motivate this work. First, fixed-resolution primitive expansion is difficult to reconcile with the spatial heterogeneity of a working face. In open regions, short fixed rollouts produce redundant nodes; in narrow regions, long fixed rollouts may exceed the geometric width of the navigable space and cause expansion deadlock. Second, exhaustive primitive expansion evaluates many low-value candidates at every node, which increases collision-checking cost for AMT with large swept areas. Third, conventional Hybrid A* often terminates once a feasible Reeds–Shepp connection to the goal is found, although the first feasible analytical connection may still contain unnecessary reversing, gear switching, or steering correction. To address these issues, this paper proposes an adaptive bounded Hybrid A* planner that introduces local-geometry-aware top-K motion primitive scheduling, adaptive rollout-length assignment, progress-reward-based dual-cost correction, and bounded continuation after the first feasible Reeds–Shepp closure. Unlike previous methods that only refine sampling resolution or improve terminal connection, the proposed planner reshapes the search process according to local geometric descriptors and separates first-solution discovery from final-path refinement for AMT planning at open-pit mine working faces.

## 3. Framework Overview

This section formulates the AMT path planning problem at open-pit mine working faces and presents the overall framework of the proposed adaptive bounded Hybrid A* planner. The proposed method assumes a known static working face map and focuses on low-speed spatial path generation in the Cartesian frame. The goal is to generate a collision-free and kinematically feasible path that satisfies the terminal pose requirement while reducing inefficient search expansion and unnecessary maneuvering cost.

### 3.1. Problem Formulation

**Figure 2:**
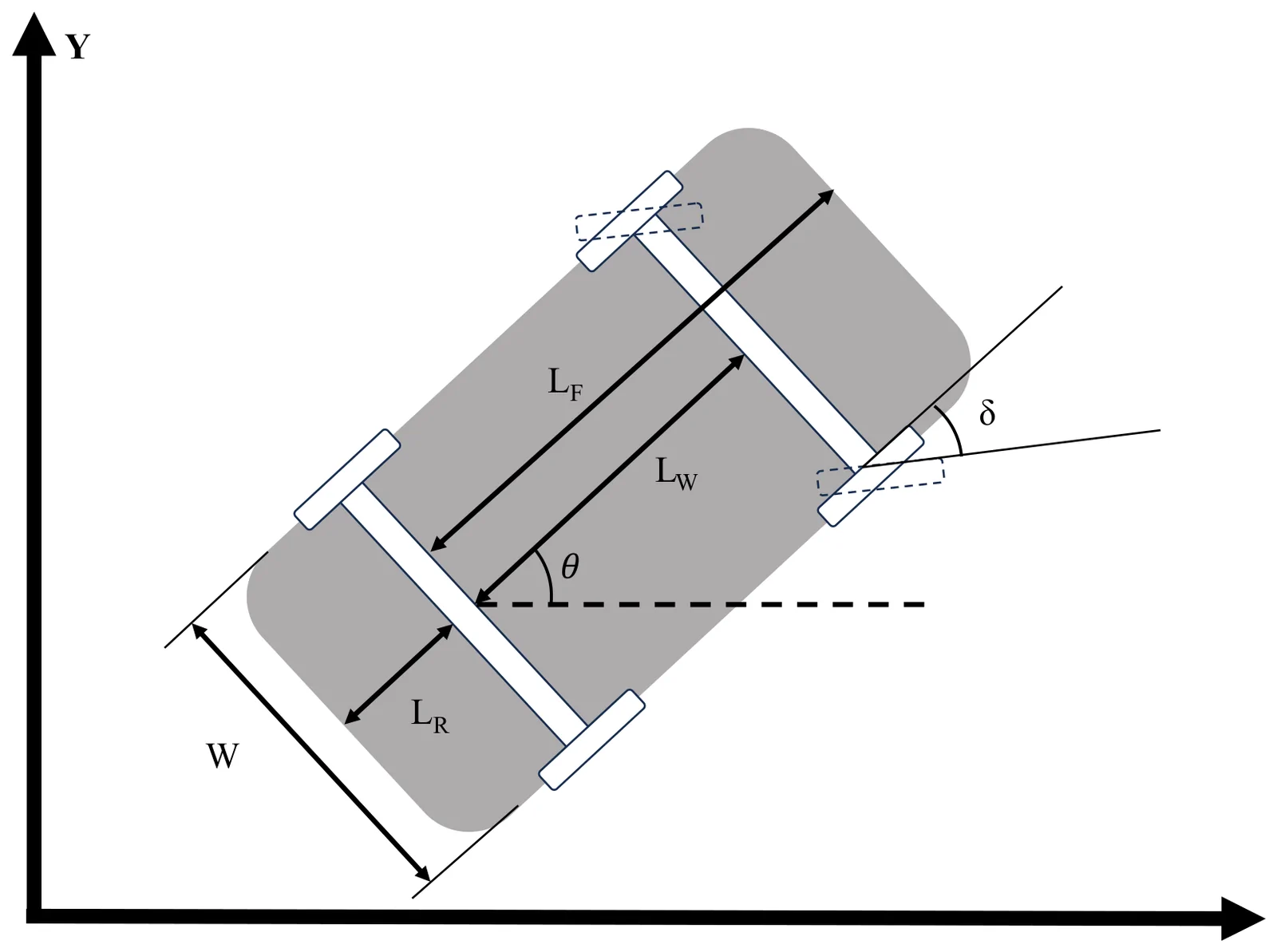
AMT kinematic model and geometric parameters.

Given a working face map M, an initial pose $x_{s}$, and a target pose $x_{g}$, the planning task is to generate a feasible path for an AMT. Since AMT maneuvering at the working face is usually performed at low speed, the vehicle is modeled by a kinematic bicycle model. The vehicle state and control input are defined as

(1) $$x={\left[x,y,\theta \right]}^{T},\;u={\left[v,\delta \right]}^{T},$$

where *x* and *y* denote the position of the rear-axle reference point in the global Cartesian frame, θ denotes the heading angle, *v* is the longitudinal velocity, and δ is the front-wheel steering angle. The low-speed kinematic model is written as

(2) $$\left\{\begin{array}{c} \dot{x}=v\cos\theta ,\\ \dot{y}=v\sin\theta ,\\ \dot{\theta }=\frac{v}{L_{W}}\tan\delta , \end{array}\right.$$

where $L_{W}$ is the wheelbase. The steering angle satisfies $\left|\delta \right|\leq \delta_{\max}$. The AMT body is represented by a rectangular envelope attached to the rear-axle reference point, as shown in Figure 2.

Let O denote the obstacle region in the working face map, and let C(x) denote the occupied region of the AMT at state x. A path is defined as

(3) $$\Pi =\{x_{0},x_{1},\ldots ,x_{N}\}.$$

The path planning problem is formulated as

(4) $$\begin{array}{cc} \min_{\Pi ,U}\; & J(\Pi )\\ s.t.\; & x_{0}=x_{s},\\ \; & x_{N}\in X_{g},\\ \; & x_{k+1}=f\left(x_{k},u_{k}\right),\;k=0,\ldots ,N-1,\\ \; & C(x_{k})\cap O=\emptyset ,\;k=0,\ldots ,N,\\ \; & |\delta_{k}|\leq \delta_{\max},\;k=0,\ldots ,N-1, \end{array}$$

where $X_{g}$ is the terminal tolerance region around the target pose, and U is the sequence of motion primitives used to generate the path.

The path cost evaluates the maneuver quality of the AMT. Considering the operational requirements at the working face, the cost function penalizes path length, reverse motion, gear switching, steering magnitude, and steering variation:

(5) $$\begin{array}{c} J\left(\Pi \right)=\sum_{k=1}^{N}[\omega_{l}l_{k}+\omega_{r}I\left(d_{k}=-1\right)l_{k}+\omega_{g}I\left(d_{k}\neq d_{k-1}\right)\\ +\omega_{s}l_{k}|\delta_{k}|+\omega_{\Delta s}l_{k}\left|\delta_{k}-\delta_{k-1}\right|], \end{array}$$

where $l_{k}$ is the traveled length of the *k*-th transition, $d_{k}\in \left\{+1,-1\right\}$ denotes the driving direction, and I(·) is the indicator function. The coefficients $\omega_{l}$, $\omega_{r}$, $\omega_{g}$, $\omega_{s}$, and $\omega_{\Delta s}$ are non-negative weights.

**Figure 3:**
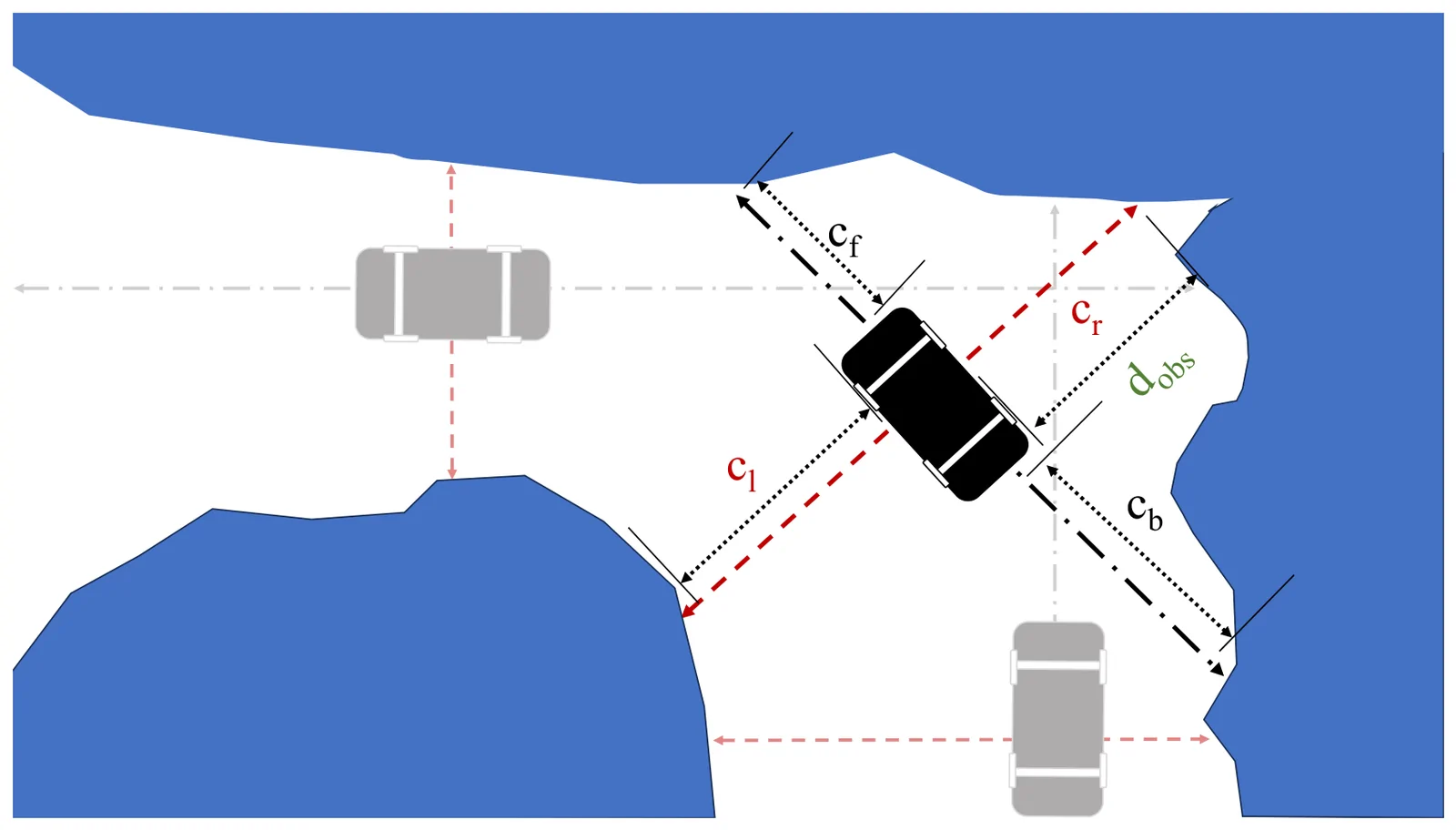
Local geometric descriptors extracted around the current AMT state.

To support local-geometry-aware expansion, a compact set of local geometric descriptors is extracted at each search node. The nearest-obstacle distance is defined as

(6) $$d_{\mathrm{obs}}\left(x\right)=\min_{o\in O}{∥p\left(x\right)-o∥}_{2},$$

where $p\left(x\right)={\left[x,y\right]}^{T}$ is the vehicle reference position. In the local vehicle frame, probing rays are cast in the forward, backward, left, and right directions to obtain the directional clearances $c_{f}$, $c_{b}$, $c_{l}$, and $c_{r}$, as illustrated in Figure 3. The lateral free width is

(7) $$w_{\mathrm{free}}=c_{l}+c_{r}.$$

The forward and backward maneuver margins are defined as

(8) $$m_{f}=\min\left(w_{\mathrm{free}}-W,c_{f}\right),$$

(9) $$m_{b}=\min\left(w_{\mathrm{free}}-W,c_{b}\right),$$

where *W* is the AMT width. The goal distance and heading error are defined as

(10) $$d_{g}\left(x\right)=\sqrt{{\left(x-x_{g}\right)}^{2}+{\left(y-y_{g}\right)}^{2}},$$

(11) $$e_{\theta }\left(x\right)=\left|\mathrm{wrap}\left(\theta -\theta_{g}\right)\right|.$$

Accordingly, the local descriptor set is written as

(12) $$F\left(x\right)=\left\{d_{\mathrm{obs}},c_{f},c_{b},c_{l},c_{r},w_{\mathrm{free}},m_{f},m_{b},d_{g},e_{\theta }\right\}.$$

The descriptor set F(x) provides the geometric basis for primitive scheduling and adaptive rollout-length assignment in the proposed planner. These ray-based clearances are used only for primitive scheduling and rollout-length assignment; collision feasibility is still verified independently using the rectangular AMT footprint along each primitive rollout.

### 3.2. Algorithm Framework

**Figure 4:**
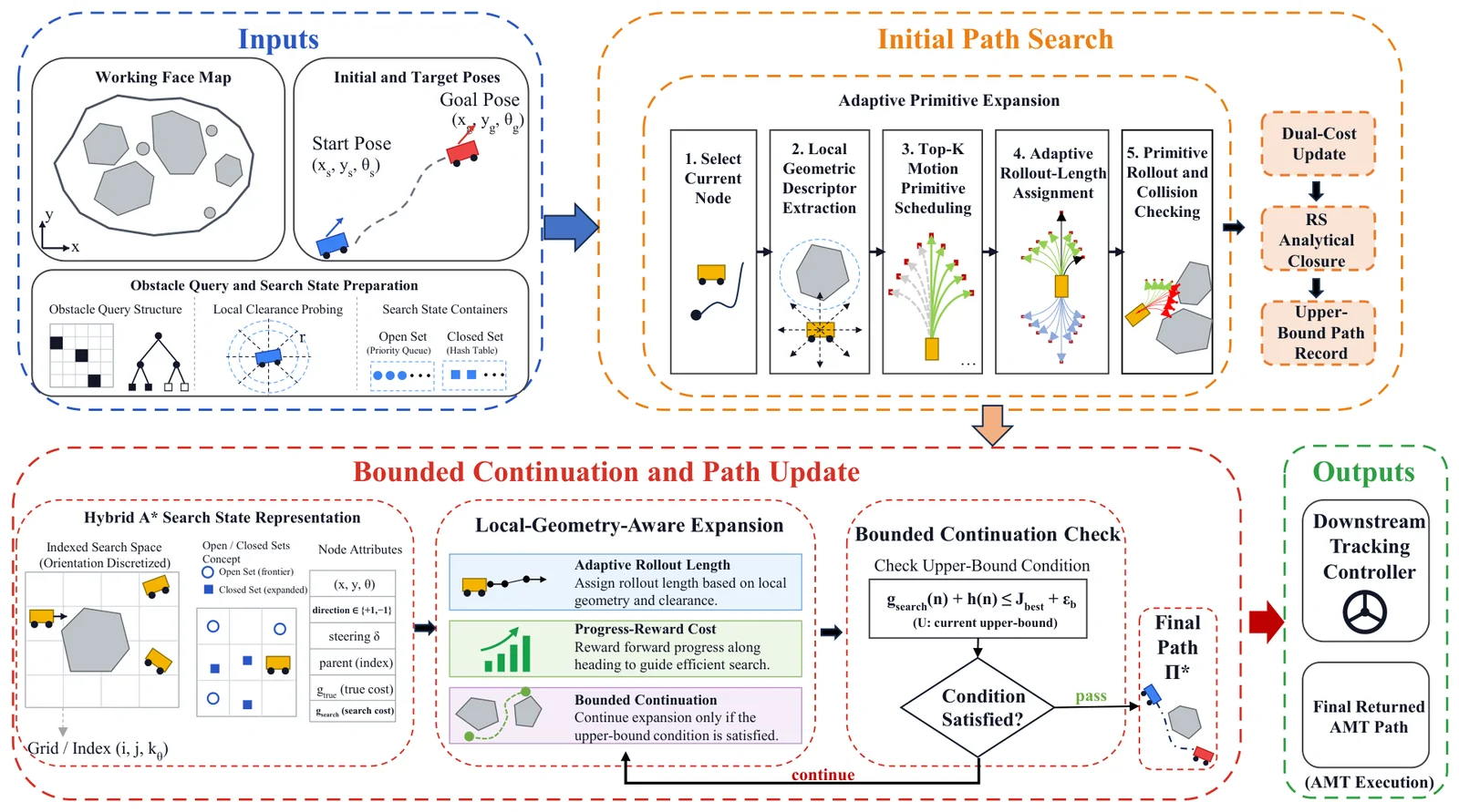
Overall framework of the proposed adaptive bounded Hybrid A* planner for AMT path planning at open-pit mine working faces.

The proposed planner is built upon the Hybrid A* search framework but modifies the primitive expansion, cost update, and termination logic. Figure 4 shows the overall architecture of the proposed adaptive bounded Hybrid A* planner. The inputs include the working face map, AMT parameters, initial pose, target pose, obstacle query structure, and search-state containers. The output is the final returned AMT path for downstream execution.

The planner first initializes the Hybrid A* search state, including the indexed search space, open set, closed set, and node attributes. At each iteration, the planner selects the current node from the open set according to the search priority. Then, the local descriptor set F(x) is extracted from the current AMT state. Based on these descriptors, the fixed motion primitive pool is ranked and filtered through a local-geometry-aware top-K scheduling strategy. This step reduces ineffective successor generation and lowers the effective branching factor.

For each selected primitive, the planner assigns an adaptive rollout length according to local obstacle proximity, direction-dependent maneuver margin, and primitive curvature. The primitive is then rolled out under a fixed integration step, and the generated candidate node is checked using the AMT rectangular envelope. If the candidate node is valid, the planner updates two costs: the true path cost and the search-ordering cost. The true path cost evaluates the final returned path, whereas the search-ordering cost introduces a progress reward to reduce the local comparison bias caused by variable-length rollout.

In parallel with primitive expansion, the planner attempts Reeds–Shepp (RS) analytical closure from selected nodes to the target pose. Unlike the baseline Hybrid A*, the proposed planner does not terminate immediately after the first feasible RS closure. Instead, the first feasible RS-connected path is recorded as an initial upper-bound candidate. The planner then continues bounded search and updates the upper-bound path whenever a lower-cost feasible candidate is found. The search terminates when the remaining nodes in the open set do not show sufficient potential to improve the current upper bound.

The framework therefore consists of four coupled mechanisms: local-geometry-aware primitive scheduling, adaptive rollout-length assignment, progress-reward-based dual-cost update, and bounded continuation after the first feasible RS closure. These mechanisms jointly reshape the Hybrid A* search process for AMT path planning in confined and geometrically irregular working face environments.

## 4. Proposed Adaptive Bounded Hybrid A* Planner

This section presents the core modules of the proposed adaptive bounded Hybrid A* planner. The planner is built upon a conventional Hybrid A* search structure, but modifies its primitive expansion, rollout scale, cost update, and termination strategy. The objective is to reduce redundant search expansion while improving the final returned path for AMT maneuvering at open-pit mine working faces.

### 4.1. Local-Geometry-Aware Top-K Motion Primitive Scheduling

In the baseline Hybrid A* planner, a fixed motion primitive pool is expanded at every search node. Each primitive is defined by a steering angle and a driving direction. Let the primitive pool be

(13) $$U={\left\{u_{i}=\left(\delta_{i},d_{i}\right)\right\}}_{i=1}^{N_{u}},$$

where $\delta_{i}$ is the steering angle and $d_{i}\in \left\{+1,-1\right\}$ denotes forward or reverse motion. In the baseline planner, all primitives in U are rolled out uniformly. This strategy is simple, but inefficient in confined working face environments because many primitives are locally irrelevant or immediately lead to collision.

**Figure 5:**
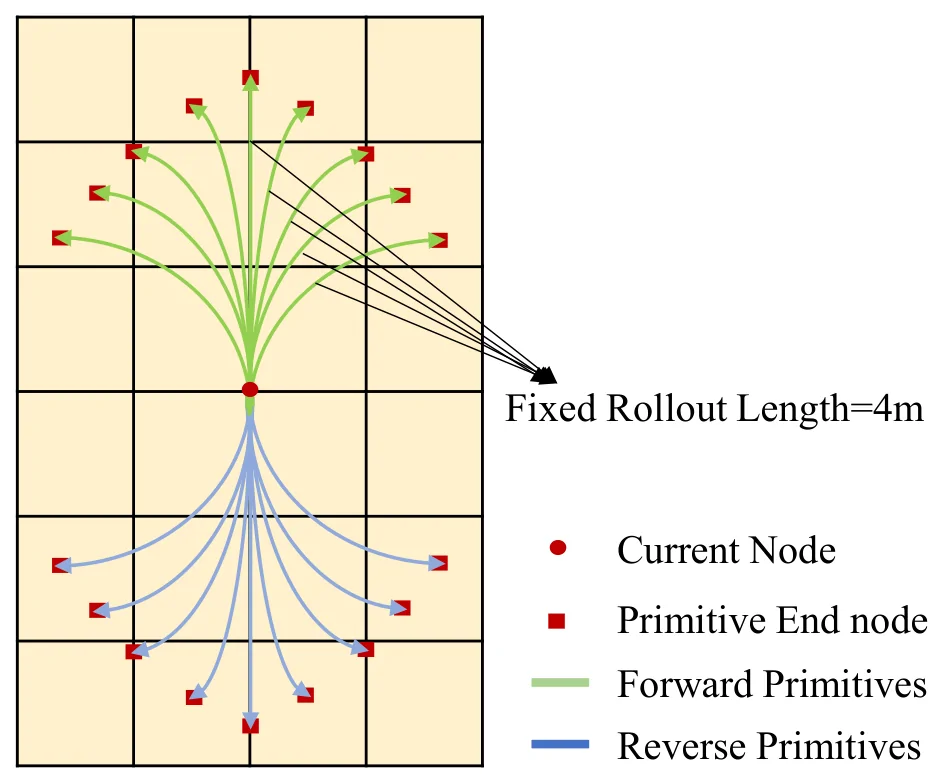
Baseline Hybrid A* primitive expansion with a fixed rollout length.

Different from the baseline expansion shown in Figure 5, the proposed planner performs local-geometry-aware primitive scheduling. The key idea is to rank the fixed primitive pool according to the local descriptor set F(x) defined in Section 3. The proposed method does not generate new control primitives online; instead, it adaptively schedules and filters the existing primitive pool.

For a primitive *u* at node x, the score is defined as

(14) $$S\left(u|x\right)=\lambda_{1}\phi_{\mathrm{dir}}\left(u,x\right)+\lambda_{2}\phi_{\mathrm{align}}\left(u,x\right)+\lambda_{3}\phi_{\mathrm{clear}}\left(u,x\right)+\lambda_{4}\left|\delta \left(u\right)\right|,$$

where $\phi_{\mathrm{dir}}$ is the directional preference term, $\phi_{\mathrm{align}}$ is the goal-alignment term, $\phi_{\mathrm{clear}}$ is the clearance-constraint term, and |δ(u)| penalizes unnecessary large steering. A smaller score indicates a higher scheduling priority.

The directional preference term is constructed according to the forward and backward maneuver margins:

(15) $$\phi_{\mathrm{dir}}\left(u,x\right)=\left\{\begin{array}{cc} -\eta_{f}, & d\left(u\right)=+1,\;m_{f}\geq m_{b},\\ -\eta_{b}, & d\left(u\right)=-1,\;m_{b}>m_{f},\\ 0, & \mathrm{otherwise}. \end{array}\right.$$

This term favors the direction with larger maneuver margin. For example, if the forward margin is larger than the backward margin, forward primitives receive better scores.

The goal-alignment term is defined as

(16) $$\phi_{\mathrm{align}}\left(u,x\right)=\left\{\begin{array}{cc} -\eta_{a}, & e_{\theta }\left(x\right)<\theta_{\mathrm{th}},\;d\left(u\right)=+1,\;\left|\delta \left(u\right)\right|<\delta_{\mathrm{th}},\\ 0, & \mathrm{otherwise}. \end{array}\right.$$

When the AMT heading is already close to the target heading, this term prioritizes small-steering forward primitives and avoids unnecessary aggressive maneuvers.

The clearance-constraint term suppresses primitives that steer toward insufficient lateral clearance:

(17) $$\phi_{\mathrm{clear}}\left(u,x\right)=\left\{\begin{array}{cc} \eta_{c}\left(c_{\mathrm{th}}-c_{l}\right), & \delta \left(u\right)>0,\;c_{l}<c_{\mathrm{th}},\\ \eta_{c}\left(c_{\mathrm{th}}-c_{r}\right), & \delta \left(u\right)<0,\;c_{r}<c_{\mathrm{th}},\\ 0, & \mathrm{otherwise}. \end{array}\right.$$

Thus, left-turn primitives are penalized when the left clearance is insufficient, and right-turn primitives are penalized when the right clearance is insufficient.

The coefficients $\eta_{f}>0$, $\eta_{b}>0$, $\eta_{a}>0$, and $\eta_{c}>0$ are user-defined positive scaling constants. Specifically, $\eta_{f}$ and $\eta_{b}$ control the magnitude of the forward- and reverse-direction preferences, respectively; $\eta_{a}$ determines the strength of the small-steering forward preference when the AMT heading is close to the target heading; and $\eta_{c}$ controls the penalty applied to primitives steering toward insufficient lateral clearance. Their numerical values are reported later in the parameter sensitivity analysis in Section 5.4.

After all primitives are scored, they are sorted in ascending order. The top-K candidate primitive set is then obtained as

(18) $$U_{K}\left(x\right)=\mathrm{TopK}\left(U,S(\cdot |x)\right).$$

To avoid collapsing the selected set into a single driving direction, a direction-diversity compensation mechanism is used. If $U_{K}\left(x\right)$ contains only forward primitives or only reverse primitives, the best-scored primitive from the opposite direction is added to the candidate set. This preserves basic maneuver diversity while reducing the effective branching factor.

### 4.2. Adaptive Rollout-Length Assignment

The baseline Hybrid A* planner uses a fixed rollout length for all motion primitives. Such a fixed expansion scale is not suitable for working face environments, where open areas and narrow regions may coexist in the same task. A short fixed rollout increases redundant node generation in open regions, whereas a long fixed rollout may cause collision or expansion deadlock in confined regions.

To address this scale mismatch, the proposed planner assigns an adaptive rollout length to each selected primitive according to the local environment and primitive curvature. The curvature of primitive *u* is defined as

(19) $$\kappa \left(u\right)=\frac{\tan\delta (u)}{L_{W}}.$$

Since the available space for forward and reverse motions may be different, the direction-dependent maneuver margin is defined as

(20) $$m_{\mathrm{dir}}\left(u,x\right)=\left\{\begin{array}{cc} m_{f}, & d(u)=+1,\\ m_{b}, & d(u)=-1. \end{array}\right.$$

The adaptive rollout length is then given by

(21) $$\begin{array}{c} L_{u}\left(x\right)=\\ \mathrm{clip}\left(L_{\min},L_{\max},\beta_{1}d_{\mathrm{obs}}\left(x\right)+\beta_{2}m_{\mathrm{dir}}\left(u,x\right)+\frac{\beta_{3}}{\left|\kappa \left(u\right)\right|+\epsilon_{\kappa }}\right), \end{array}$$

where $L_{\min}$ and $L_{\max}$ are the minimum and maximum rollout lengths, $\beta_{1}$, $\beta_{2}$, and $\beta_{3}$ are weighting coefficients, and $\epsilon_{\kappa }$ is a small positive constant.

**Figure 6:**
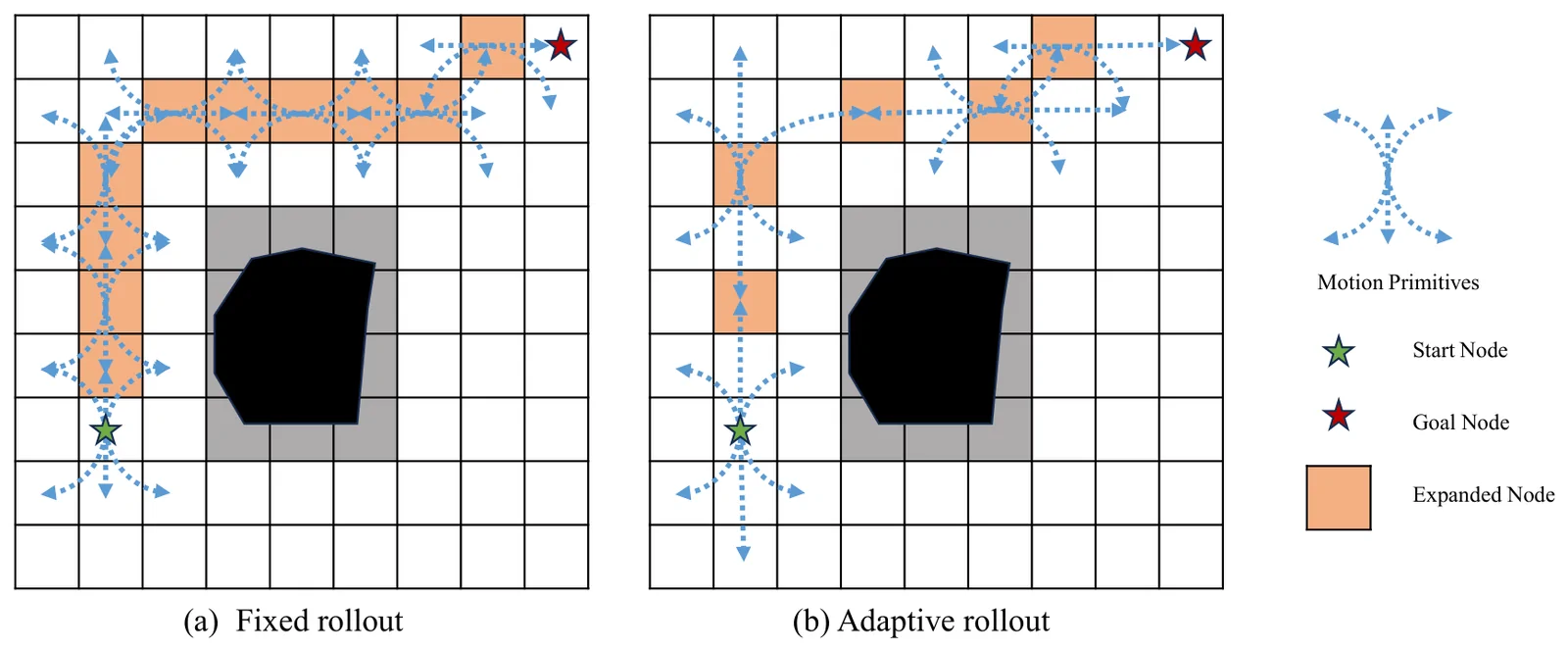
Illustration of node generation under different rollout-length strategies. (**a**) Fixed rollout length produces dense expansion nodes. (**b**) Adaptive rollout length reduces node density while preserving feasible progress.

As shown in Figure 6, the rollout length becomes longer in open areas and shorter near constrained regions. The term $d_{\mathrm{obs}}$ reflects general obstacle proximity, $m_{\mathrm{dir}}$ reflects direction-dependent maneuverability, and |κ(u)| suppresses overly long high-curvature expansion. Therefore, the proposed rollout strategy reduces node density in open regions while maintaining finer maneuver resolution in confined areas.

It should be emphasized that the proposed method does not change the integration step used in primitive rollout. Let Δs denote the fixed integration step. The number of integration steps for primitive *u* is

(22) $$N_{u}\left(x\right)=\left\lceil \frac{L_{u}\left(x\right)}{\Delta s}\right\rceil .$$

Therefore, the proposed strategy is a variable-length primitive expansion method under a fixed integration step, rather than a variable integration-step method. This design preserves collision-checking resolution while allowing the expansion scale to adapt to local spatial conditions.

### 4.3. Progress-Reward-Based Dual-Cost Update

Adaptive rollout length changes the cost-comparison basis among successor nodes. In the baseline Hybrid A* planner, all primitives have the same rollout length, so their incremental costs are compared on a similar spatial scale. Under variable-length rollout, however, short primitives may appear artificially cheaper simply because they travel a shorter distance. This may cause the planner to prefer local small-step maneuvers rather than primitives that make effective progress toward the target.

To reduce this bias, the proposed planner separates the true path cost from the search-ordering cost. The true path cost evaluates the physical cost of the final returned path. For a child node *n* generated from its parent node par(n), the true incremental cost is

(23) $$\Delta g_{\mathrm{true}}\left(n\right)=J_{\mathrm{len}}\left(n\right)+J_{\mathrm{rev}}\left(n\right)+J_{\mathrm{gear}}\left(n\right)+J_{\mathrm{steer}}\left(n\right)+J_{\Delta \mathrm{steer}}\left(n\right).$$

The true path cost is recursively updated as

(24) $$g_{\mathrm{true}}\left(n\right)=g_{\mathrm{true}}\left(\mathrm{par}\left(n\right)\right)+\Delta g_{\mathrm{true}}\left(n\right).$$

To guide the search ordering under variable-length rollout, a progress reward is introduced. The progress made by node *n* is defined as

(25) $$R_{\mathrm{prog}}\left(n\right)=\max\left(0,d_{g}\left(\mathrm{par}\left(n\right)\right)-d_{g}\left(n\right)\right).$$

To avoid excessive reward caused by long rollouts, the reward is clipped:

(26) $${\bar{R}}_{\mathrm{prog}}\left(n\right)=\min\left(R_{\mathrm{prog}}\left(n\right),R_{\max}^{\mathrm{prog}}\right).$$

Although $R_{\mathrm{prog}}$ is already indirectly bounded by the maximum rollout length $L_{\max}$, the clipping is retained as a saturation mechanism for search ordering rather than for mathematical boundedness; it prevents long rollouts from receiving disproportionate progress rewards while leaving the true path cost unchanged.

The search-ordering cost is then defined as

(27) $$g_{\mathrm{search}}\left(n\right)=g_{\mathrm{true}}\left(n\right)-\lambda_{p}{\bar{R}}_{\mathrm{prog}}\left(n\right),$$

where $\lambda_{p}$ is the progress-reward weight. The node priority used in the open set is

(28) $$f\left(n\right)=g_{\mathrm{search}}\left(n\right)+h\left(n\right),$$

where h(n) denotes the heuristic cost.

The progress reward is only used for node ordering and is not included in the final path cost. Thus, the final returned path is still evaluated by $g_{\mathrm{true}}$, while $g_{\mathrm{search}}$ improves the fairness of local comparison under variable-length expansion.

### 4.4. Bounded Continuation After the First Feasible RS Closure

In the baseline Hybrid A* planner, the search usually terminates once a collision-free Reeds–Shepp (RS) connection from the current node to the target pose is found. This early-termination strategy can quickly produce a feasible path, but the first feasible RS-connected path may not be the best complete path. In confined working-face scenarios, early RS closure may be reached from a node whose preceding path contains unnecessary reversing, gear switching, or steering correction.

To address this issue, the proposed planner treats the first feasible RS closure as an initial upper-bound candidate rather than an immediate final result. Suppose that node *n* can be connected to the target pose $x_{g}$ through a collision-free RS curve. The candidate complete-path cost is

(29) $$J_{\mathrm{cand}}\left(n\right)=g_{\mathrm{true}}\left(n\right)+J_{\mathrm{RS}}\left(n,x_{g}\right),$$

where $J_{\mathrm{RS}}\left(n,x_{g}\right)$ is the true cost of the RS connection from node *n* to the target pose. If no feasible complete path has been recorded, the upper bound is initialized as

(30) $$J_{\mathrm{best}}\leftarrow J_{\mathrm{cand}}\left(n\right).$$

The corresponding complete path is stored as the current best path $\Pi^{*}$.

Unlike the baseline method, the proposed planner continues the search after the first feasible RS closure. For any subsequent node $n^{\prime }$, if its RS-connected candidate path satisfies

(31) $$J_{\mathrm{cand}}\left(n^{\prime }\right)<J_{\mathrm{best}},$$

then the upper bound and the best path are updated:

(32) $$J_{\mathrm{best}}\leftarrow J_{\mathrm{cand}}\left(n^{\prime }\right),\;\Pi^{*}\leftarrow \Pi_{\mathrm{cand}}\left(n^{\prime }\right).$$

By continuing the search under the current upper bound, the planner may find a lower-cost complete path. The bounded continuation process terminates when the best-ranked node in the open set no longer has sufficient potential to improve the current upper bound:

(33) $$\min_{n\in \mathrm{OPEN}}\left(g_{\mathrm{search}}\left(n\right)+h\left(n\right)\right)>J_{\mathrm{best}}+\varepsilon_{b},$$

where $\varepsilon_{b}\geq 0$ is a continuation tolerance. Because $g_{\mathrm{search}}$ contains the progress-reward term, Equation (33) is used as a heuristic stopping criterion rather than as an optimality-preserving lower-bound test.

### 4.5. Algorithm Procedure

The complete procedure of the proposed adaptive bounded Hybrid A* planner is summarized in Algorithm 1.
**Algorithm 1** Adaptive Bounded Hybrid A* Planner**Require:** Working face map M, start pose $x_{s}$, target pose $x_{g}$, AMT parameters, primitive pool U
**Ensure:** Final returned path $\Pi^{*}$
  1:Initialize OPEN, CLOSED, $J_{\mathrm{best}}\leftarrow +\infty$, $\Pi^{*}\leftarrow Ø$  2:Insert the start node into OPEN  3:**while** OPEN is not empty **do**  4: Select $n=\arg\min_{m\in \mathrm{OPEN}}\left(g_{\mathrm{search}}\left(m\right)+h\left(m\right)\right)$  5: Remove *n* from OPEN and insert it into CLOSED  6: Extract local descriptor set $F(x_{n})$  7: Attempt RS analytical closure from *n* to $x_{g}$  8: **if** the RS connection is collision-free **then**  9:  Compute $J_{\mathrm{cand}}\left(n\right)=g_{\mathrm{true}}\left(n\right)+J_{\mathrm{RS}}\left(n,x_{g}\right)$10:  **if** $J_{\mathrm{cand}}\left(n\right)<J_{\mathrm{best}}$ **then**11:   Update $J_{\mathrm{best}}\leftarrow J_{\mathrm{cand}}\left(n\right)$12:   Update $\Pi^{*}\leftarrow \Pi_{\mathrm{cand}}\left(n\right)$13:  **end if**14: **end if**15: Compute $U_{K}\left(x_{n}\right)=\mathrm{TopK}\left(U,S\left(\cdot |x_{n}\right)\right)$16: Apply direction-diversity compensation to $U_{K}\left(x_{n}\right)$ if necessary17: **for** each primitive $u\in U_{K}\left(x_{n}\right)$ **do**18:  Compute adaptive rollout length $L_{u}\left(x_{n}\right)$ using (21)19:  Roll out primitive *u* from node *n* under fixed integration step Δs20:  Generate child node $n^{\prime }$21:  **if** $n^{\prime }$ is valid and collision-free **then**22:   Compute $\Delta g_{\mathrm{true}}\left(n^{\prime }\right)$23:   Update $g_{\mathrm{true}}\left(n^{\prime }\right)$24:   Compute $R_{\mathrm{prog}}\left(n^{\prime }\right)$ and ${\bar{R}}_{\mathrm{prog}}\left(n^{\prime }\right)$25:   Compute $g_{\mathrm{search}}\left(n^{\prime }\right)$26:   Insert or update $n^{\prime }$ in OPEN27:  **end if**28: **end for**29: **if** $J_{\mathrm{best}}<+\infty$ and $\min_{m\in \mathrm{OPEN}}\left(g_{\mathrm{search}}\left(m\right)+h\left(m\right)\right)>J_{\mathrm{best}}+\varepsilon_{b}$ **then**30:  **break**31: **end if**32:**end while**33:**return** $\Pi^{*}$


## 5. Experimental Evaluation and Discussion

This section evaluates the proposed adaptive bounded Hybrid A* planner from four aspects: mechanism-level effectiveness, parameter sensitivity, planning performance in abstract mine-like scenarios, and planning performance in real-map-derived mining working-face scenarios. Both first-solution and final-solution results are reported to distinguish initial feasible-path discovery from subsequent refinement.

### 5.1. Experimental Setup

All experiments are conducted in static unstructured environments represented by occupancy-grid maps. The AMT is modeled by the kinematic bicycle model defined in Section 3.1, and collision checking is performed using the rectangular vehicle envelope.

All planners were implemented in Python 3.14 and executed on the same computer equipped with an AMD Ryzen 5 5600H processor (Advanced Micro Devices, Inc., Santa Clara, CA, USA) at 3.30 GHz and 16 GB of RAM. The search maps use a Cartesian occupancy-grid resolution of 2.0 m and a heading discretization of 15°. Obstacle-boundary points are sampled at an approximate spatial interval of 0.4 m. The common primitive propagation/motion step used in the reported comparisons is 1.0 m. The maximum distance used for local clearance probing is 20 m.

The baseline uses a conventional Hybrid A* search front end with a fixed rollout length, full primitive-pool expansion, and conventional path-cost ordering. The proposed planner uses the same vehicle model, primitive pool, map representation, heuristic configuration, collision-checking resolution, and start–target poses, but introduces local-geometry-aware top-*K* primitive scheduling, adaptive rollout-length assignment, and progress-reward-based dual-cost ordering.

To provide an external comparison with a recent improved Hybrid A* strategy, an improved Hybrid A* planner (IHA*) is additionally implemented for the abstract mine-like scenarios. The implemented IHA* follows the first-stage planning framework in [9]: a two-dimensional A* search is first used to obtain a coarse collision-free guidance path, intermediate poses are extracted according to the heading variation of the coarse path, and conventional Hybrid A* is subsequently used to connect adjacent intermediate poses. Only the path-search stage is considered in this study; the subsequent trajectory-optimization stage of the original method is not included, because the present work focuses on spatial path planning rather than trajectory optimization.

For fairness, IHA* uses the same AMT geometry, occupancy-grid maps, start–target poses, steering limit, Reeds–Shepp turning-radius constraint, and rectangular-footprint collision checker as the baseline and proposed planners. The complete path returned by IHA* is also re-evaluated using the common true path-cost function defined in Equation (5). The proposed adaptive primitive scheduling, adaptive rollout, dual-cost ordering, and bounded continuation mechanisms are not incorporated into IHA*.

For a fair internal comparison, the baseline and proposed search front ends use the same multi-RS continuation procedure. Throughout Section 5, the term *baseline* therefore refers to conventional Hybrid A* combined with this common continuation procedure.

The tested scenarios include three groups. The first group consists of simplified validation maps used to isolate the effect of each proposed module. The second group consists of abstract mine-like scenarios that reproduce typical constrained maneuvering patterns, including terminal docking, bottleneck traversal, diagonal obstacle avoidance, L-shaped transfer, and converging-corridor alignment. The third group consists of real-map-derived mining working-face scenarios, which contain irregular obstacle boundaries and working-face-like spatial structures. These scenarios are used to examine whether the proposed planner remains effective beyond simplified synthetic maps.

Table 1 lists the main AMT model and planner parameters. The values should be kept identical for the baseline and the proposed planner unless otherwise stated. This setting ensures that the performance difference comes from the proposed search strategy rather than from changes in vehicle geometry, map resolution, or collision-checking resolution.

### 5.2. Evaluation Metrics

The evaluation metrics are divided into first-solution metrics and final-solution metrics. For both the baseline and proposed planners, the first-solution metrics are recorded when the first collision-free RS-connected complete path is obtained. The final-solution metrics are recorded when the common multi-RS continuation stage terminates. Thus, the first-solution metrics characterize initial feasible-path discovery, whereas the final-solution metrics characterize the best incumbent path returned after evaluating multiple feasible RS connections.

The planning time measures computational efficiency. The number of expanded nodes reflects the depth and breadth of the actual search process. The number of generated nodes measures the total successor-generation burden and is closely related to collision-checking cost. The number of RS attempts reflects the frequency of analytical closure trials. The path cost is calculated using the true cost function defined in Section 3 and includes path length, reverse-motion penalty, gear-switching penalty, steering penalty, and steering-change penalty. The evaluation metrics are summarized in Table 2.

This metric design is important for the proposed method. A planner may obtain a feasible path quickly but return a higher-cost trajectory if it terminates too early. Therefore, first-solution performance and final-returned-path performance are analyzed separately.

For the additional comparison with IHA*, only planning success, planning time, and common path cost are compared. For the baseline and proposed planners, the first complete feasible solution is used, whereas for IHA*, the complete path returned after the intermediate-point-guided search is used. Expanded nodes, generated nodes, and RS attempts are not used for the three-planner comparison because IHA* combines a two-dimensional A* guidance stage with multiple segmented Hybrid A* searches, and therefore its node-count statistics are not directly equivalent to those of the single-stage Hybrid A* search front ends.

### 5.3. Mechanism-Level Validation

This subsection validates the three main behavioral changes introduced by the proposed planner: adaptive rollout-length assignment, top-K primitive scheduling, and bounded continuation after the first feasible RS closure. These experiments are performed on simplified validation maps to make the effect of each module visually interpretable. The dual-cost update is not treated as an independent mechanism-level switch because it is introduced specifically to compensate for the cost-comparison bias caused by variable-length rollout, as explained in Section 4.3.

#### 5.3.1. Effect of Adaptive Rollout-Length Assignment

**Figure 7:**
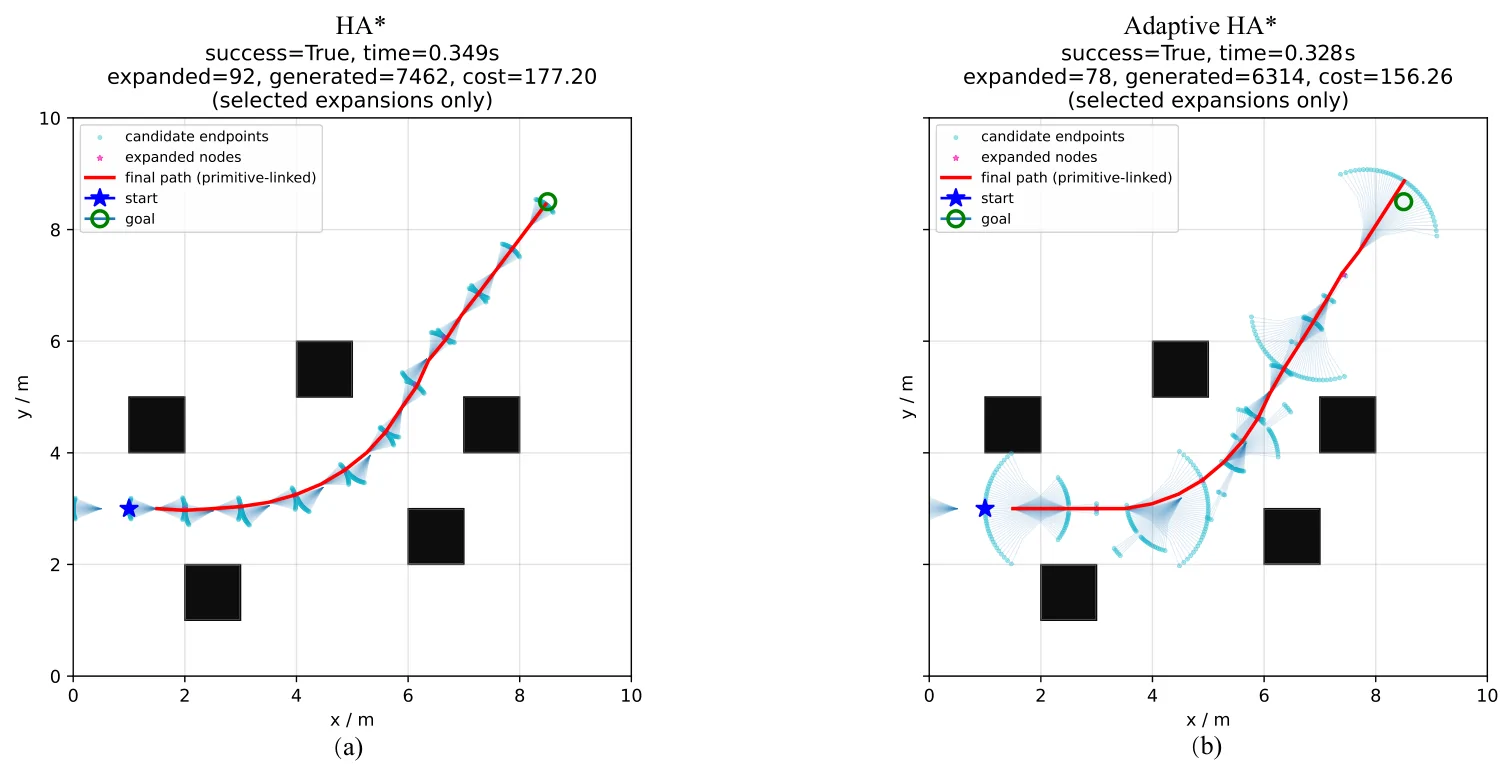
Mechanism-level validation of adaptive rollout-length assignment: (**a**) baseline Hybrid A* with fixed rollout length; (**b**) Hybrid A* with adaptive rollout-length assignment. Black squares denote obstacles in both panels.

Figure 7 compares the baseline Hybrid A* planner and the planner with adaptive rollout-length assignment. Under fixed rollout length, the baseline planner generates dense and redundant search branches even in locally open regions. After adaptive rollout is introduced, the planner uses longer rollouts in open regions and shorter rollouts near constrained areas. This result shows that adaptive rollout reduces the mismatch between a fixed primitive length and local free-space geometry.

The main effect of adaptive rollout is not only acceleration. It changes the spatial scale of expansion according to local geometry. In open regions, the planner reduces node density by using longer rollouts. In narrow regions, the planner preserves maneuver resolution by shortening high-risk expansions. However, variable-length rollout also changes the local cost-comparison basis, which motivates the dual-cost update introduced in Section 4.

#### 5.3.2. Effect of Top-K Primitive Scheduling

**Figure 8:**
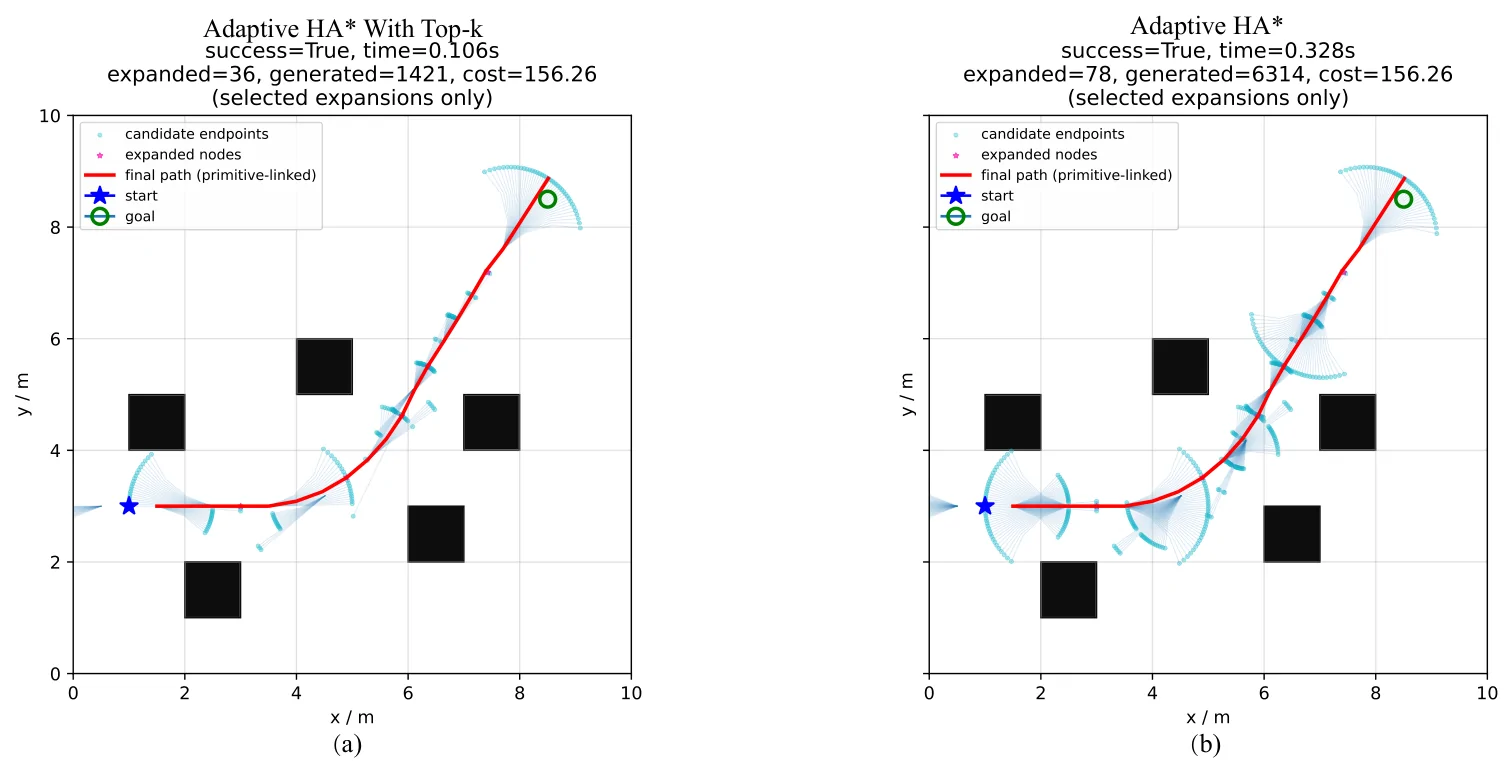
Mechanism-level validation of local-geometry-aware top-K primitive scheduling: (**a**) adaptive Hybrid A* with top-K primitive scheduling; (**b**) adaptive Hybrid A* without top-K filtering. Black squares denote obstacles in both panels.

Figure 8 compares the search behavior with and without top-K primitive scheduling. Without top-K filtering, the planner still evaluates many primitives that are unlikely to contribute to feasible or efficient maneuvers under the current local geometry. After top-K scheduling is enabled, the search tree becomes more concentrated, and redundant successor generation is reduced.

The role of top-K scheduling is to reduce the effective branching factor. It should not be interpreted as a direct path-quality improvement mechanism. Its benefit comes from suppressing locally irrelevant primitive rollouts and reducing unnecessary collision checks. Since top-K scheduling is heuristic, the proposed planner preserves direction diversity to avoid selecting only forward or only reverse primitives.

#### 5.3.3. Effect of Bounded Continuation After First RS Closure

**Figure 9:**
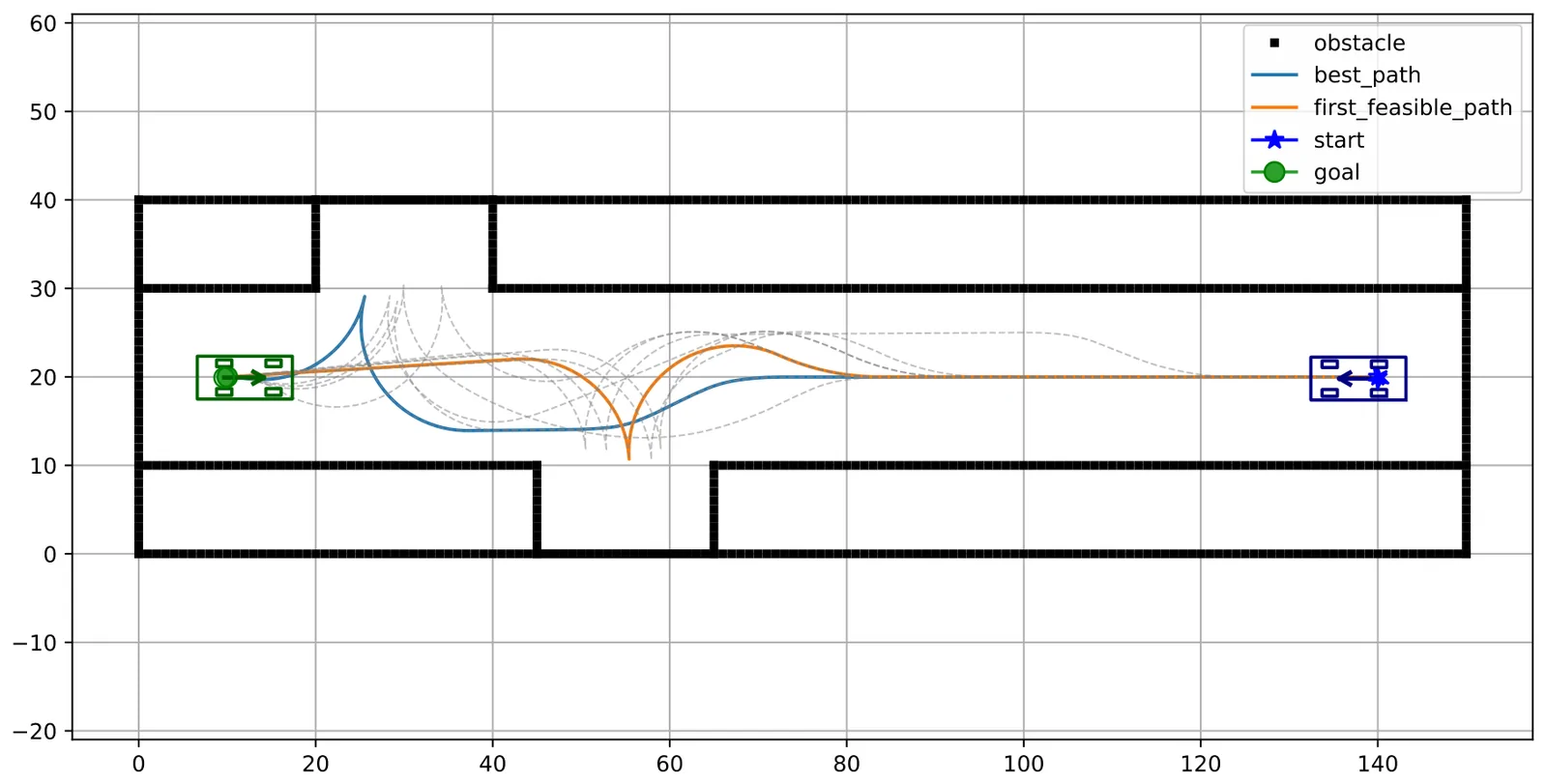
Mechanism-level validation of bounded continuation after the first feasible RS closure.

Figure 9 illustrates the role of bounded continuation after the first feasible RS closure. In the first-solution evaluation mode, the first collision-free RS-connected path is recorded immediately when it is found. During the subsequent multi-RS continuation stage, this first feasible path is retained as the initial incumbent solution, while additional feasible RS connections are evaluated. If a later RS-connected candidate has a lower true cost, the incumbent path is updated. The same first-solution recording and multi-RS continuation procedure is applied to both the baseline and proposed search front ends.

This example shows that the first feasible RS-connected path is not necessarily the best complete path. In confined working face environments, early RS closure may occur at a node whose preceding path contains unnecessary reversing or steering correction. Bounded continuation changes the role of RS closure from an immediate stopping condition to an upper-bound update mechanism.

### 5.4. Parameter Sensitivity Analysis

This subsection analyzes the sensitivity of the proposed planner to the adaptive rollout coefficients and the top-K parameter. The purpose is not to identify a single-metric optimum, but to select a stable default setting that balances search efficiency and final path quality.

In addition to the parameters varied below, Table 3 summarizes the main fixed coefficients and activation thresholds used in the proposed planner.

The parameters in Table 3 are determined in three ways. The rollout coefficients $\beta_{1}$ and $\beta_{2}$ and the retained primitive number *K* are selected according to the sensitivity analyses in this section. Vehicle-scale activation thresholds and the remaining scheduling and cost coefficients are fixed through preliminary calibration and are kept unchanged for all reported scenarios. The small constant $\epsilon_{\kappa }$ is used only for numerical regularization.

The adaptive rollout length in Section 4 depends mainly on $\beta_{1}$, $\beta_{2}$, and $\beta_{3}$. The coefficient $\beta_{1}$ controls the influence of nearest-obstacle distance, $\beta_{2}$ controls the influence of direction-dependent maneuver margin, and $\beta_{3}$ regularizes the effect of primitive curvature. Since $\beta_{1}$ and $\beta_{2}$ directly determine how rollout length responds to local geometry, the sensitivity analysis focuses on $\beta_{1}$ and $\beta_{2}$, while $\beta_{3}$ is fixed at its nominal value.

Because planning time, generated nodes, expanded nodes, and final cost differ substantially across scenario cases, raw metrics are not directly averaged. Instead, case-wise normalized metrics are used. For a scenario–case pair *c* and a tested parameter configuration *p*, let m(c,p) denote a metric value. The case-specific minimum is

(34) $$m_{\min}\left(c\right)=\min_{p\in P}m\left(c,p\right).$$

The normalized value is

(35) $$\tilde{m}\left(c,p\right)=\frac{m(c,p)}{m_{\min}\left(c\right)}.$$

The final normalized result is averaged across all cases:

(36) $$\bar{m}\left(p\right)=\frac{1}{\left|C\right|}\sum_{c\in C}\tilde{m}\left(c,p\right),$$

where C is the set of scenario–case pairs and P is the set of parameter configurations. A normalized value larger than one indicates degradation relative to the best configuration within the same case.

**Figure 10:**
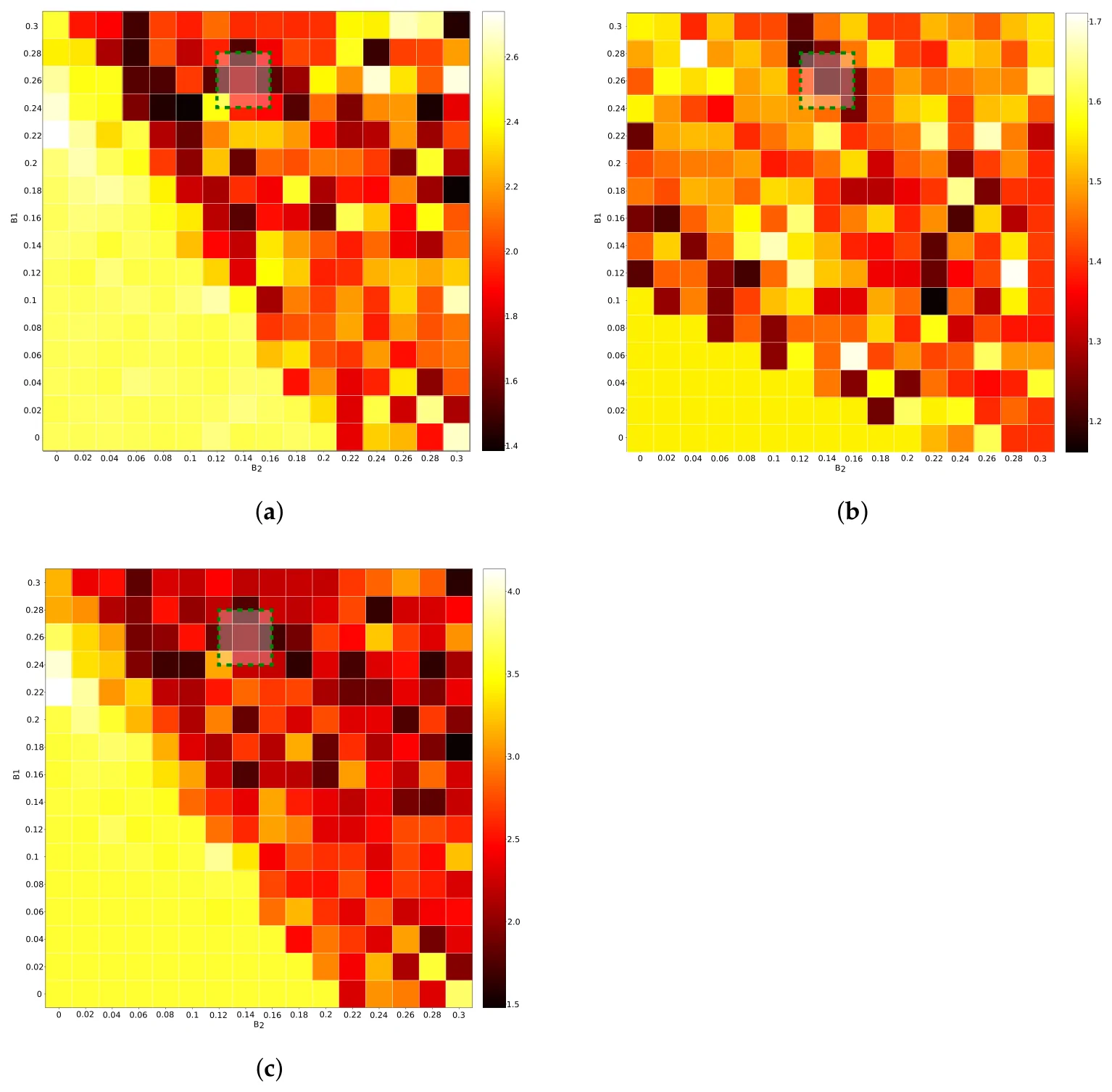
Sensitivity heatmaps of $\beta_{1}$ and $\beta_{2}$ with respect to normalized planning time, normalized final path cost, and normalized generated nodes. (**a**) Heatmap of average planning time, (**b**) heatmap of average final cost, and (**c**) heatmap of average generated nodes.

Figure 10 shows the normalized heatmaps for planning time, generated nodes, and final path cost with respect to $\beta_{1}$ and $\beta_{2}$. The heatmaps for planning time and generated nodes show similar patterns, indicating that runtime is mainly affected by search-tree size. Increasing $\beta_{1}$ from a small value to a moderate value generally reduces generated nodes because longer rollouts are used in open regions. The effect of $\beta_{2}$ is more coupled: moderate values help exploit direction-dependent maneuver space, whereas excessive values may introduce local bias.

The final path cost heatmap does not fully overlap with the minimum-time region. This result indicates that the most efficient parameter setting does not necessarily produce the lowest-cost path. Therefore, $\beta_{1}$ and $\beta_{2}$ should be selected as an efficiency–quality trade-off rather than as a single-metric optimum.

By jointly considering the three heatmaps, it can be concluded that the favorable combinations of $\beta_{1}$ and $\beta_{2}$ are not concentrated at a single isolated optimum, but rather distributed over a relatively stable low-value region. In the present experiments, a recommended parameter region can be approximately identified as $\beta_{1}\in \left[0.24,\;0.28\right]$, $\beta_{2}\in \left[0.12,\;0.16\right]$.

This region is selected by the green dotted box, as shown in Figure 10. This region simultaneously achieves low normalized planning time, low normalized generated nodes, and comparatively low normalized final cost. Since the goal of parameter selection is to determine a robust default setting rather than a single-metric extreme point, the final default configuration is selected near the center of this favorable region as $\beta_{1}=0.26$, $\beta_{2}=0.14$.

**Figure 11:**
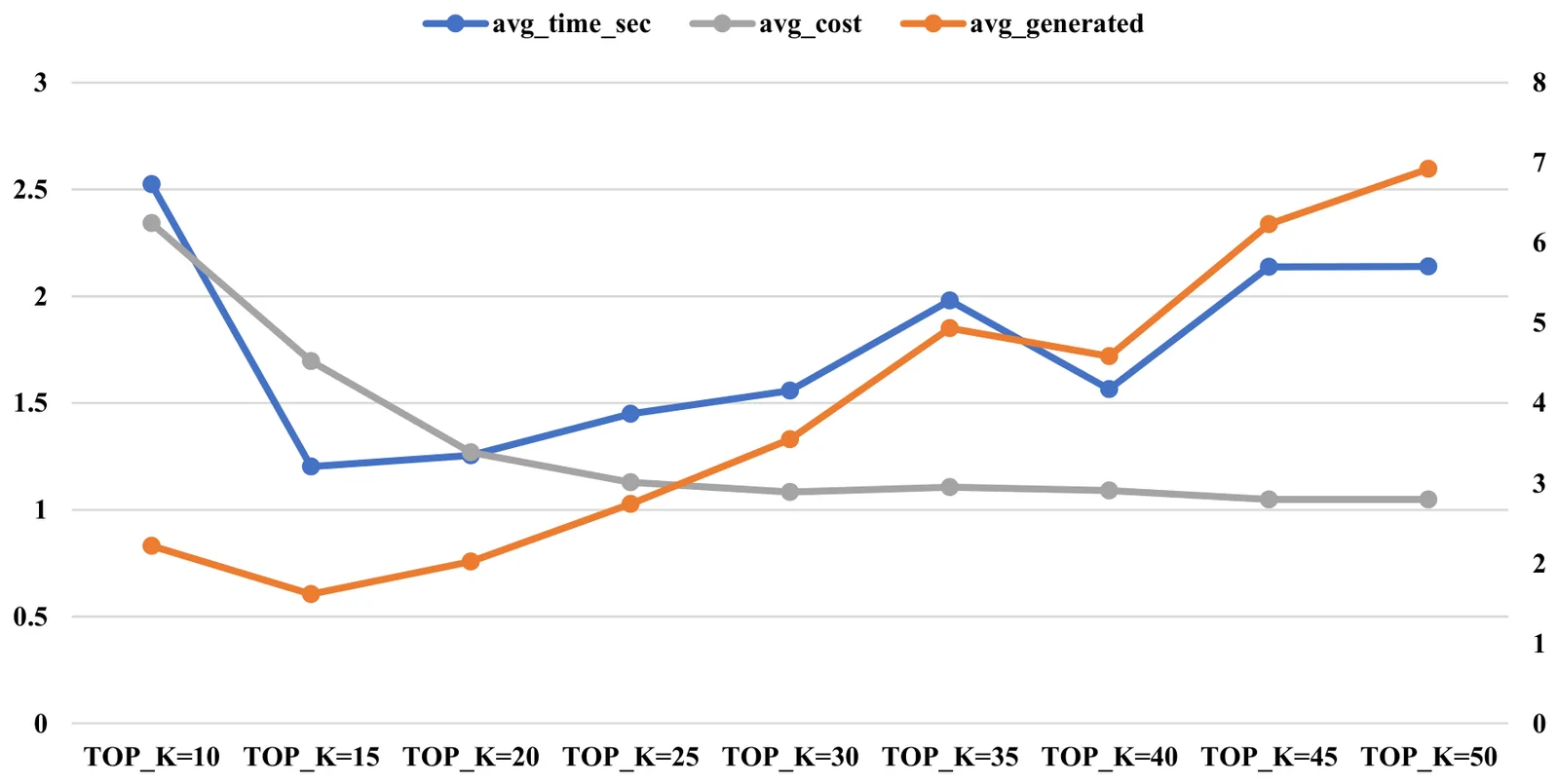
Sensitivity of top-K with respect to normalized planning time, normalized final path cost, and normalized generated nodes. The normalized generated nodes are plotted on the secondary vertical axis.

Figure 11 shows the effect of top-K on normalized planning time, final path cost, and generated nodes. The number of generated nodes increases as top-K increases because more primitives are retained at each node. The final path cost decreases rapidly when top-K increases from a very small value, but it gradually saturates after reaching a moderate range. This trend indicates that small top-K values may over-prune useful primitives, whereas overly large values mainly increase computation without substantial path-quality gain. In the present experiments, a favorable trade-off region can be identified approximately as top-*K* ∈ [20, 25].

Within this interval, the normalized final cost has already dropped close to its plateau, while the normalized planning time and normalized generated nodes remain comparatively low. Therefore, the final default setting used in the subsequent experiments is chosen as top-*K* = 25.

This choice avoids the over-pruning effect observed at very small top-*K* values while also preventing the substantial search-scale inflation that occurs when top-*K* becomes too large.

Based on the sensitivity results, the default parameters are selected from a stable trade-off region rather than from an isolated optimum. This choice avoids over-aggressive rollout, over-pruning of primitives, and excessive search-tree inflation.

### 5.5. Results in Abstract Mine-like Scenarios

**Figure 12:**
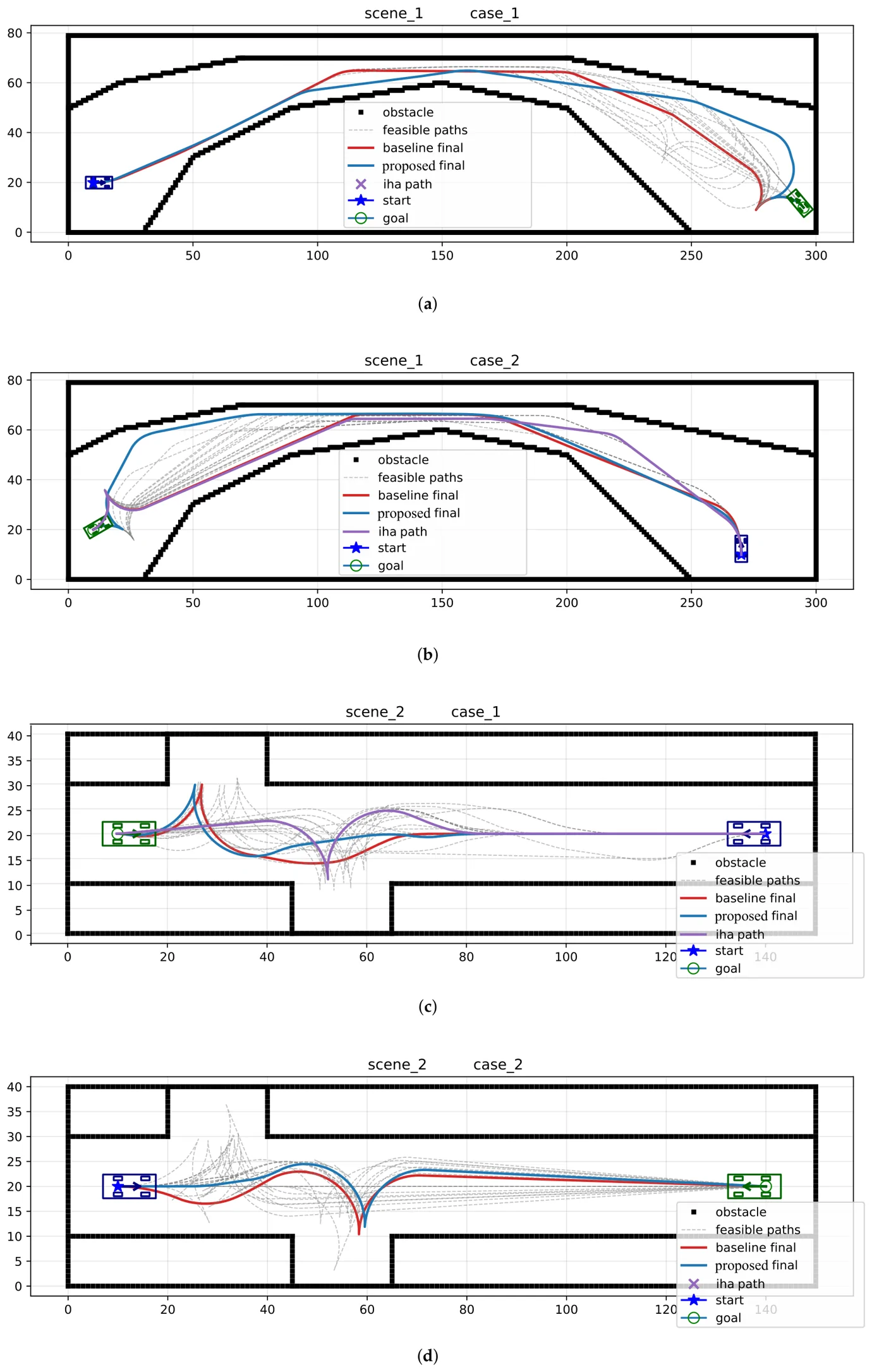
Path comparison in abstract mine-like scenarios: (**a**) scene_1 case_1, long-corridor terminal docking; (**b**) scene_1 case_2, long-corridor terminal docking; (**c**) scene_2 case_1, bottleneck-constrained through-corridor planning; (**d**) scene_2 case_2, bottleneck-constrained through-corridor planning. No IHA* path is shown in (**a**) or (**d**) because IHA* did not obtain a complete feasible solution in these cases.

**Figure 13:**
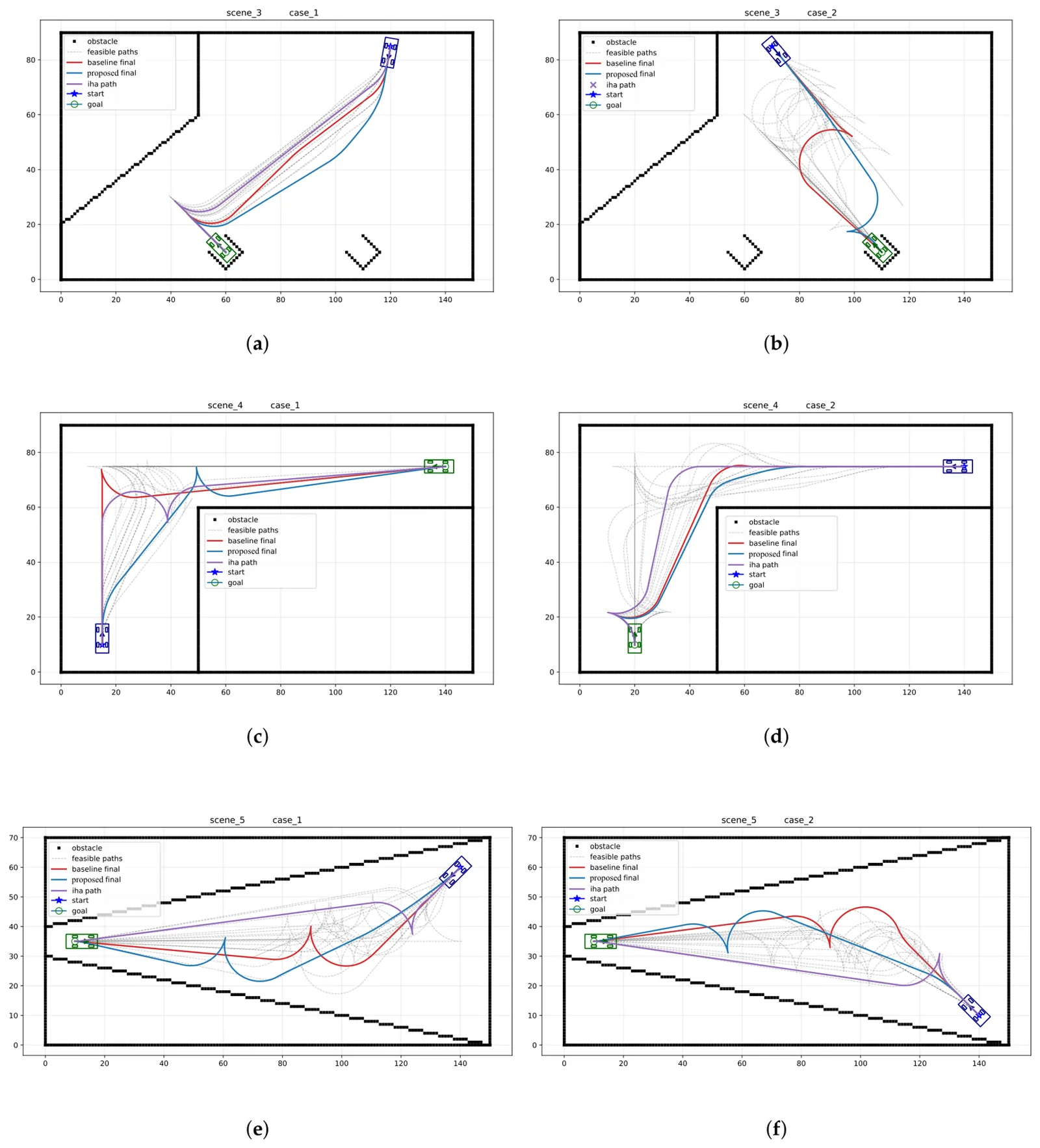
Path comparison in abstract mine-like scenarios: (**a**) scene_3 case_1, diagonal traversal; (**b**) scene_3 case_2, diagonal traversal; (**c**) scene_4 case_1, L-shaped transfer; (**d**) scene_4 case_2, L-shaped transfer; (**e**) scene_5 case_1, converging-corridor multi-stage alignment; (**f**) scene_5 case_2, converging-corridor multi-stage alignment. No IHA* path is shown in (**b**) because IHA* did not obtain a complete feasible solution in this case.

The proposed planner was first evaluated in five abstract mine-like scenarios, which represent typical AMT maneuvering patterns at working faces, including long-corridor terminal docking, bottleneck traversal, diagonal obstacle avoidance, L-shaped transfer, and converging-corridor alignment, as shown in Figure 12 and Figure 13. These scenarios are used both to evaluate the proposed local-geometry-aware expansion and bounded continuation mechanisms against the baseline Hybrid A* planner and to provide an external comparison with IHA*. Each scenario contains two start–goal cases, resulting in a total of ten planning tasks.

Table 4 summarizes the average final-solution results in the five abstract mine-like scenarios. The reported final solutions are obtained after applying the same multi-RS continuation procedure to both the conventional and proposed search front ends. Compared with the baseline Hybrid A* planner, the proposed planner reduces the average final planning time from 20.72 s to 9.38 s, corresponding to a 54.73% reduction. The average number of expanded nodes decreases from 2080 to 1078, and the average number of RS attempts decreases from 104.0 to 53.9, both corresponding to a 48.17% reduction. The most significant improvement is observed in generated nodes, which decrease from 170,478 to 44,157, corresponding to a 74.10% reduction. This result confirms that local-geometry-aware top-K scheduling effectively reduces successor generation and collision-checking burden.

In terms of final path cost, the average cost decreases from 1835.29 to 1601.04, corresponding to a 12.76% reduction. The improvement is scenario-dependent. In scene_1, the proposed planner reduces final planning time by 80.67%, expanded nodes by 79.22%, generated nodes by 89.62%, RS attempts by 79.22%, and final cost by 21.79%. This indicates that the proposed expansion strategy is particularly effective when the baseline planner suffers from substantial redundant exploration. In scene_2, final planning time, generated nodes, and final cost are reduced by 39.75%, 64.20%, and 4.43%, respectively, showing that the method still improves efficiency in bottleneck-constrained planning, although the path-quality gain is more moderate.

Scene_3 provides a useful non-dominance case. The proposed planner reduces generated nodes by 42.77% and final cost by 25.77%, but final planning time increases by 5.60%, while expanded nodes and RS attempts increase by 14.44%. Scene_5 shows a similar partial trade-off: generated nodes and final cost decrease by 44.97% and 18.20%, respectively, but expanded nodes and RS attempts increase by 10.00%. These cases indicate that reducing successor generation does not always guarantee fewer expanded nodes or shorter runtime, because the remaining search branches may still require difficult collision checking or RS closure attempts.

Table 5 reports the corresponding first-solution results in the same abstract mine-like scenarios. These metrics are recorded when each search front end obtains its first collision-free RS-connected complete path, before the subsequent multi-RS continuation stage. Compared with the baseline planner, the proposed planner reduces average first-solution time from 10.22 s to 4.26 s, corresponding to a 58.30% reduction. The average first expanded nodes decrease from 966 to 456, and first RS attempts decrease from 48.3 to 22.8, both corresponding to a 52.80% reduction. The average first generated nodes decrease from 79,130 to 18,655, corresponding to a 76.42% reduction. These results indicate that the proposed planner can substantially accelerate the discovery of the first feasible complete path.

The first-solution table reveals an important difference between feasibility discovery and final path refinement. In scene_1, the proposed planner reduces first-solution time by 86.81%, generated nodes by 93.59%, and first-solution cost by 15.28%, showing a clear improvement in both efficiency and first feasible path quality. However, in scene_2 and scene_3, the proposed planner requires more time and more expanded nodes to obtain the first feasible path. Specifically, first-solution time increases by 117.95% in scene_2 and 106.60% in scene_3, while first expanded nodes increase by 168.09% and 150.00%, respectively. Nevertheless, these two cases do not contradict the final-solution improvement because bounded continuation and subsequent RS candidate updates can still reduce the final returned cost.

The cost behavior is more revealing. Although the proposed planner reduces first-solution cost by 15.28% in scene_1 and 4.34% in scene_2, it increases first-solution cost by 14.21%, 8.17%, and 13.49% in scene_3, scene_4, and scene_5, respectively. As a result, the average first-solution cost increases by 4.32%. However, the average final path cost decreases by 12.76%. This contrast confirms that the proposed method should not be evaluated only by the first feasible path. The first feasible RS closure provides an initial upper-bound candidate, while the final returned path is improved through bounded continuation.

#### Comparison with IHA*

Using the common setup and evaluation protocol defined in Section 5.1 and Section 5.2, IHA* is compared with the baseline and proposed planners on the same ten start–goal tasks in the five abstract mine-like scenarios. All successful paths are evaluated using the common true path-cost function defined in Equation (5).

Table 6 summarizes the comparison. Both the baseline and proposed planners successfully solve all ten planning tasks, whereas IHA* succeeds in seven of the ten cases. IHA* fails to obtain a complete feasible path in scene_1 case_1, scene_2 case_2, and scene_3 case_2. These failures indicate that the intermediate-point decomposition based on a two-dimensional coarse path can become sensitive to the compatibility between the holonomic guidance path and the nonholonomic maneuver required by the large AMT.

For successful runs, the reported time denotes the elapsed wall-clock time required to obtain the complete feasible path. For the three failed IHA* cases, the reported time denotes the elapsed time until the planner terminated without obtaining a complete feasible solution, and the corresponding path cost is therefore not reported.

To avoid bias caused by failed cases, the quantitative time and cost comparison is further examined on the seven tasks successfully solved by all three planners. Over these common-success cases, the average planning times of the baseline, IHA*, and proposed planners are 8.54 s, 18.79 s, and 3.52 s, respectively. Thus, the proposed planner reduces the average initial feasible-path planning time by 58.79% relative to the baseline and by 81.26% relative to IHA*. This result indicates that the proposed adaptive expansion strategy retains the global Hybrid A* search structure while avoiding the additional computational burden caused by repeated intermediate-point-guided sub-searches in difficult cases.

The corresponding average path costs over the seven common-success cases are 2045.76, 3457.17, and 2154.57 for the baseline, IHA*, and proposed planners, respectively. The proposed planner reduces the average path cost by 37.68% relative to IHA*, although its initial feasible-path cost remains 5.32% higher than that of the baseline on this common-success subset. This result is consistent with the first-solution analysis in Table 5: the proposed search strategy does not necessarily produce the lowest-cost first feasible path in every case. Its main advantage is that it generates feasible solutions more reliably and generally more efficiently than IHA*, while the subsequent bounded continuation stage further improves the final returned path.

The visual comparisons in Figure 12 and Figure 13 further illustrate the difference among the three planners. IHA* can generate feasible paths efficiently when the two-dimensional coarse guidance and extracted intermediate poses are compatible with the required AMT maneuver. However, its performance deteriorates when the coarse guidance does not adequately represent the forward–reverse nonholonomic maneuver required by the large vehicle. In contrast, the proposed planner does not rely on externally generated intermediate poses and successfully solves all ten benchmark tasks while retaining the ability to refine the initial feasible path through bounded continuation.

### 5.6. Results in Real-Map-Derived Mining Working-Face Scenarios

**Figure 14:**
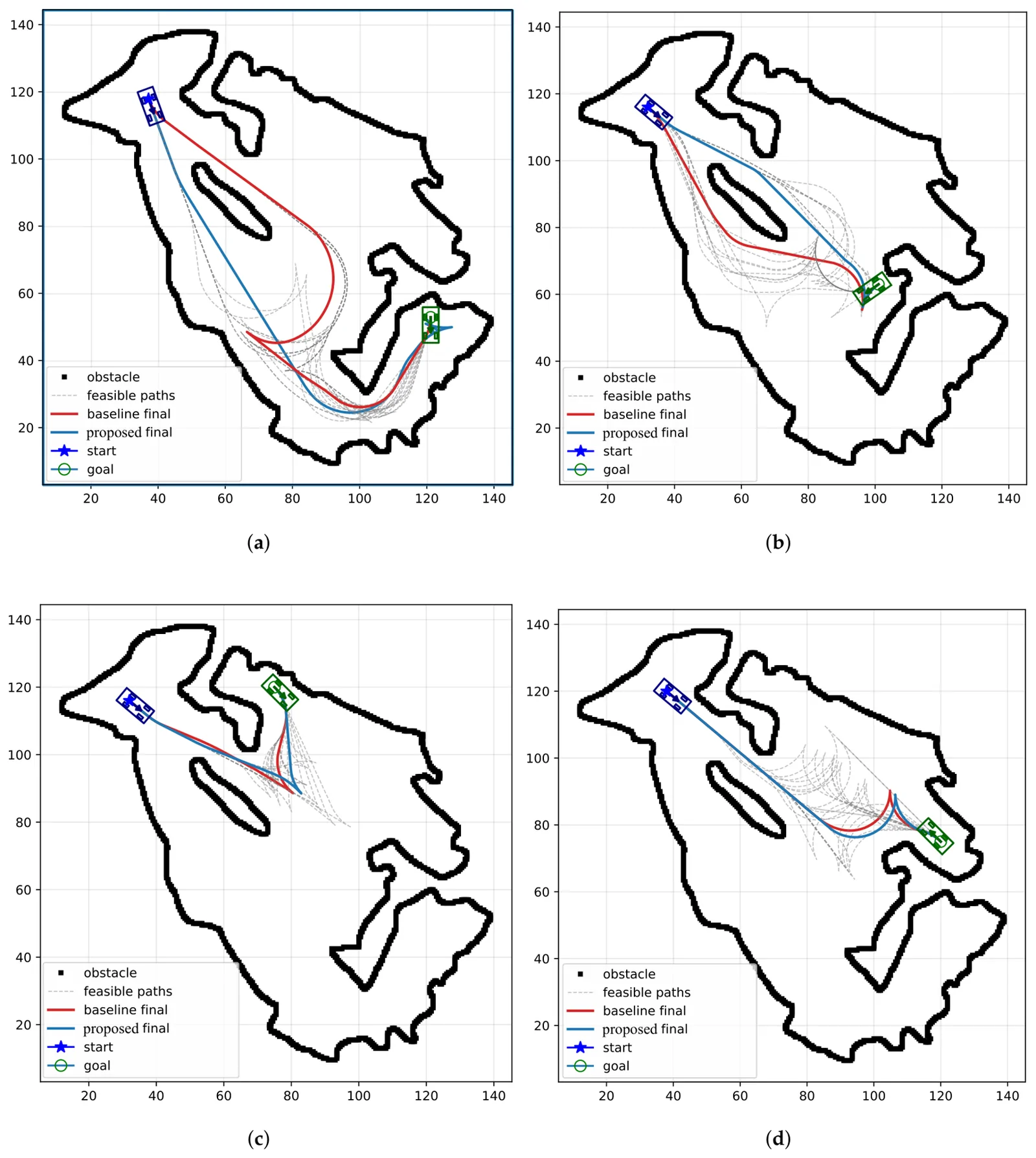
Path comparison in real-map-derived mining working-face scenarios with irregular obstacle boundaries: (**a**) case_1; (**b**) case_2; (**c**) case_3; (**d**) case_4.

The proposed planner was further evaluated in static simulations constructed from point-cloud scans of a real open-pit coal-mine working face. Obstacle and free-space boundaries were extracted from the point-cloud data and subsequently represented in occupancy-grid planning maps. These scenarios preserve non-convex obstacle outlines, local bottlenecks, and less regular free-space distributions than the abstract mine-like scenarios. The experiments are offline path-planning simulations rather than on-vehicle field tests, and are used to examine whether the proposed planner remains effective when the simplified benchmark geometry is replaced by real-map-derived working-face geometry. The experimental planning paths are shown in Figure 14.

Table 7 reports the final-solution results in the real-map-derived mining working face cases with irregular obstacle boundaries. For both planners, the reported final solutions are obtained after the same multi-RS continuation procedure. Averaged over the four cases, the proposed planner reduces final planning time from 181.52 s to 135.92 s, corresponding to a 25.12% reduction. The average number of final generated nodes decreases from 555,058 to 344,245, corresponding to a 37.98% reduction. The average final path cost decreases from 1886.53 to 1751.61, corresponding to a 7.15% reduction. The reductions in expanded nodes and RS attempts are smaller, both decreasing by 7.53%. This indicates that, in irregular working face scenarios, the primary benefit of the proposed planner is the reduction of generated successors rather than a uniform reduction of all intermediate search indicators.

The case-wise results show that final generated nodes are reduced in all four real working face cases. The reductions are 37.58%, 47.08%, 49.26%, and 32.93% from case_1 to case_4, respectively. Final planning time is also reduced in all four cases, with reductions of 25.02%, 28.39%, 33.85%, and 8.61%. Final cost decreases by 6.60%, 9.33%, 2.88%, and 10.89%, respectively. These results indicate that the proposed planner remains effective when obstacle boundaries are highly irregular.

**Figure 15:**
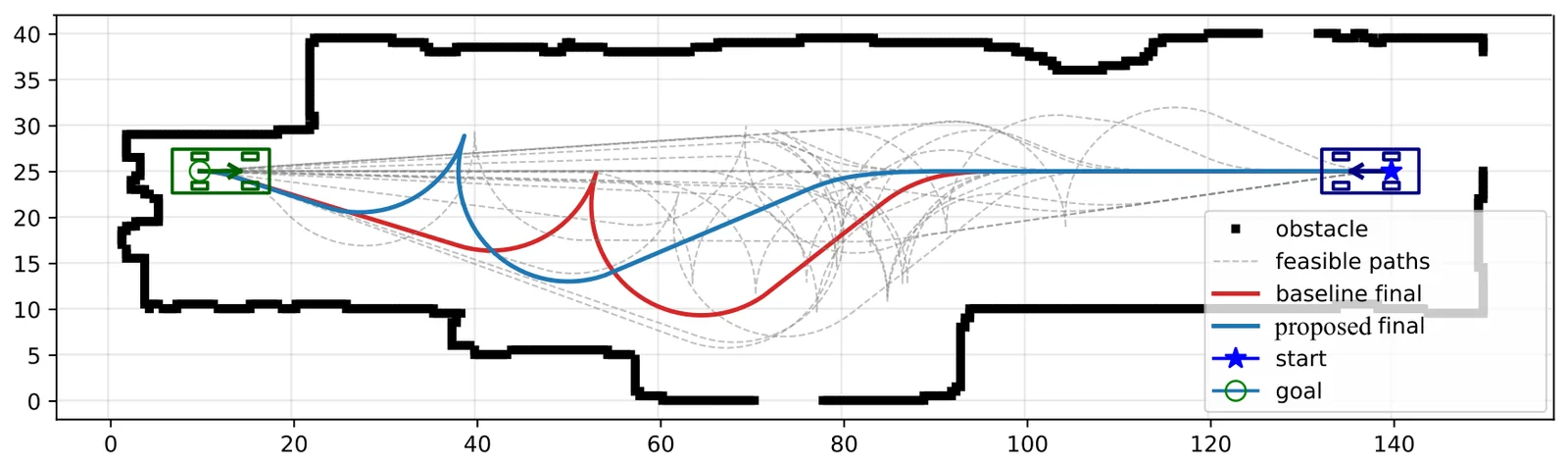
Path comparison in a real-map-derived mining working-face corridor scenario.

In addition to the irregular working face cases, a corridor-like real-map-derived mining working-face case is evaluated to examine the effect of bounded continuation in a spatially constrained environment. Figure 15 shows the path comparison, and Table 8 reports the quantitative results.

In this corridor case, the proposed planner reduces final planning time by 17.59%, final generated nodes by 35.78%, and final path cost by 24.99%. The reduction in final generated nodes again confirms that top-K scheduling and adaptive rollout reduce redundant successor generation. The improvement in final path cost is more pronounced than in the irregular working face cases, indicating that bounded continuation can be particularly useful when the first feasible connection is not sufficient to represent the best available maneuver.

The first-solution results in Table 8 reveal a different behavior. The proposed planner reduces first-solution time by 37.11%, first generated nodes by 49.91%, and first RS attempts by 25.00%, but the first-solution cost increases by 10.76%. Therefore, the first feasible path is found faster but is not better in terms of cost. After bounded continuation, however, the proposed planner reduces the path cost from 3931.33 to 1971.08, corresponding to a 49.86% improvement from its own first feasible path. By comparison, the baseline cost decreases from 3549.47 to 2627.80, corresponding to a 25.97% improvement. This comparison shows that bounded continuation contributes more substantially to the proposed planner in this corridor working face case.

### 5.7. Integrated Analysis and Limitations

The complete experimental results reveal three key observations. First, the proposed planner most consistently reduces generated nodes. In the five abstract mine-like scenarios, final generated nodes are reduced by 74.10% on average. In the real-map-derived mining working-face irregular cases, they are reduced by 37.98% on average. In the real-map-derived mining working-face corridor case, they are reduced by 35.78%. Across all reported final-solution cases, generated nodes are reduced in every case. This confirms that top-K primitive scheduling and adaptive rollout-length assignment effectively reduce redundant successor generation.

Second, final path cost is more consistently improved than first-solution cost. In the abstract mine-like scenarios, final path cost decreases by 12.76% on average, while first-solution cost increases by 4.32%. In the real-map-derived mining working-face corridor case, first-solution cost also increases by 10.76%, but final path cost decreases by 24.99%. These results show that the proposed planner should not be judged solely by the first feasible path. Because the same multi-RS continuation procedure is applied to both methods, the difference in final-path performance mainly reflects the different RS-connectable candidate states generated by the two search front ends. The proposed search strategy does not always produce a lower-cost first solution, but it can generate subsequent candidate states that lead to a lower-cost final incumbent path during the common multi-RS refinement stage.

Third, the proposed planner does not uniformly dominate the baseline in every intermediate metric. In scene_3, final generated nodes and final cost decrease, but final planning time, expanded nodes, and RS attempts increase. In scene_5, generated nodes and final cost decrease, but expanded nodes and RS attempts increase by 10.00%. This non-dominance behavior is consistent with the heuristic nature of local-geometry-aware scheduling. The proposed planner reduces low-value successor generation, but the remaining selected branches may still require difficult collision checking or RS closure attempts.

The first limitation comes from top-K primitive scheduling. A primitive that appears locally unattractive may still be useful for a globally favorable maneuver. If such a primitive is removed during top-K filtering, bounded continuation cannot recover that branch later. Direction-diversity compensation reduces this risk, but it cannot eliminate it completely. Therefore, the top-K filtering is heuristic and does not provide a formal completeness guarantee.

The second limitation comes from adaptive rollout length. Longer rollouts can reduce search depth in open regions, but they may also place the AMT in a less favorable heading and require later correction. Shorter rollouts preserve maneuver resolution in confined regions, but they may increase the number of expanded nodes. The progress-reward-based dual-cost update mitigates this bias, but it does not remove the trade-off between expansion scale and future correction cost.

The third limitation comes from the local nature of the geometric descriptors. Obstacle clearance, maneuver margin, goal distance, and heading alignment describe the immediate neighborhood of the AMT. They do not fully encode the global topology of the working face. Therefore, when a globally useful maneuver initially appears locally unfavorable, the proposed planner may lose part of its advantage.

A further limitation is that the present study assumes a known static occupancy-grid map and an accurately localized vehicle pose. Dynamic obstacles, temporal changes of the working-face boundary, incomplete map information, perception errors, localization uncertainty, and sensing latency are not explicitly modeled. In an operating mine, these factors can affect both collision checking and the validity period of a planned path. The current validation is therefore limited to offline spatial path planning, and the planner has not yet been integrated into a complete autonomous-mining-truck stack for closed-loop field operation. Future work will combine the proposed search strategy with online map updating, obstacle prediction, uncertainty-aware collision checking, trajectory tracking, and incremental replanning before conducting on-vehicle field validation.

The proposed framework is not inherently restricted to the specific AMT used in this study. For other low-speed Ackermann-steered industrial vehicles with comparable nonholonomic motion characteristics, such as yard tractors, agricultural transport vehicles, or car-like logistics vehicles, the framework can be transferred by re-parameterizing the vehicle geometry, wheelbase, steering limit, collision footprint, rollout bounds, local-clearance thresholds, and maneuver-cost weights. However, vehicles with substantially different kinematic structures, such as articulated trucks or differential-drive platforms, would require corresponding modifications to the vehicle model and motion-primitive library rather than direct parameter substitution.

Overall, the proposed planner improves the efficiency–quality trade-off of Hybrid A* for AMT path planning at open-pit mine working faces. Its most stable benefit is the reduction of generated nodes, while its final path-quality improvement is closely related to bounded continuation after the first feasible RS closure.

## 6. Conclusions

This paper proposed an adaptive bounded Hybrid A* planner for AMTs operating at open-pit mine working faces. The proposed method addresses the inefficiency of conventional Hybrid A* in confined unstructured environments by integrating local-geometry-aware top-K motion primitive scheduling, adaptive rollout-length assignment, progress-reward-based dual-cost update, and bounded continuation after the first feasible Reeds–Shepp closure. Experimental results in abstract mine-like scenarios and real-map-derived mining working-face scenarios demonstrate that the proposed planner effectively reduces redundant successor generation, improves computational efficiency, and enhances final returned path quality. In the abstract mine-like scenarios, the proposed planner reduces average final planning time by 54.73%, generated nodes by 74.10%, and final path cost by 12.76%. In the real-map-derived mining working-face scenarios with irregular obstacle boundaries, it reduces average final planning time by 25.12%, generated nodes by 37.98%, and final path cost by 7.15%. The comparison between first-solution and final-returned-path results further shows that the first feasible RS closure should not be treated as the final solution; instead, bounded continuation enables the planner to refine the initial feasible candidate and obtain a lower-cost final path. These results confirm that the proposed framework improves the efficiency–quality trade-off of Hybrid A* for AMT path planning in confined and geometrically irregular working face environments. The current validation remains limited to known static maps and offline simulation; future work will address dynamic obstacles, perception and localization uncertainty, online replanning, and closed-loop on-vehicle field validation.

## Figures and Tables

**Table 1:** AMT model and planner parameters used in the experiments.

Parameter	Value	Meaning
$L_{F}$	7.6 m	Front extent measured from the rear-axle reference point
$L_{R}$	3.0 m	Rear extent measured from the rear-axle reference point
*W*	4.77 m	AMT width
$L_{W}$	5.5 m	Wheelbase
$\delta_{\max}$	0.43 rad	Maximum steering angle
Δs	1.0 m	Primitive integration step
$L_{\min}$	2.0 m	Minimum rollout length
$L_{\max}$	8.0 m	Maximum rollout length
*K*	25	Number of selected primitives

**Table 2:** Evaluation metrics used in the experiments.

Metric	Description
First-solution time	Time to obtain the first feasible complete path
First expanded nodes	Expanded nodes before the first feasible solution
First generated nodes	Generated nodes before the first feasible solution
First RS attempts	RS closure attempts before the first feasible solution
First-solution cost	True cost of the first feasible complete path
Final planning time	Total planning time before termination
Final expanded nodes	Total expanded nodes before termination
Final generated nodes	Total generated nodes before termination
Final RS attempts	Total RS closure attempts before termination
Final path cost	True cost of the final returned path

**Table 3:** Main coefficients and thresholds used in the proposed planner.

Parameter	Value	Role
$\lambda_{1}$	1.0	Directional-preference weight
$\lambda_{2}$	1.0	Goal-heading-alignment weight
$\lambda_{3}$	1.0	Lateral-clearance weight
$\lambda_{4}$	0.2	Small-steering preference weight
$\eta_{f}$	0.8	Forward-direction preference gain
$\eta_{b}$	1.6	Reverse-direction preference gain
$\eta_{a}$	1.2	Heading-alignment gain
$\eta_{c}$	$2.2\;m^{-1}$	Lateral-clearance penalty gain
$c_{\mathrm{th}}$	3.0 m	Lateral-clearance activation threshold
$\theta_{\mathrm{th}}$	12°	Heading-alignment threshold
$\delta_{\mathrm{th}}$	8°	Small-steering threshold
*K*	25	Number of retained motion primitives
$\beta_{1}$	0.26	Obstacle-distance contribution to rollout length
$\beta_{2}$	0.14	Maneuver-margin contribution to rollout length
$\beta_{3}$	0.04	Curvature contribution to rollout length
$\epsilon_{\kappa }$	$10^{-3}\;m^{-1}$	Curvature regularization constant
$\lambda_{p}$	1.0	Progress-reward weight
$R_{\max}^{\mathrm{prog}}$	3.0 m	Progress-reward saturation limit
$\omega_{l}$	1.0	Path-length cost weight
$\omega_{r}$	40.0	Reverse-motion cost weight
$\omega_{g}$	500.0	Gear-switching cost weight
$\omega_{s}$	15.0	Steering-magnitude cost weight
$\omega_{\Delta s}$	20.0	Steering-change cost weight

**Table 4:** Average Final-Solution Results in Different Scenarios.

Scene	Mode	Average Value				
		Time (s)	Expanded	Generated	RS Attempts	Cost
scene_1	baseline	48.06	4620	378,758	231.0	1333.86
scene_1	proposed	9.29	960	39,319	48.0	1043.23
scene_2	baseline	36.93	3980	326,278	199.0	2318.47
scene_2	proposed	22.25	2850	116,809	142.5	2215.83
scene_3	baseline	9.28	900	73,718	45.0	1305.30
scene_3	proposed	9.80	1030	42,189	51.5	968.86
scene_4	baseline	6.98	700	57,318	35.0	1862.39
scene_4	proposed	3.24	330	13,489	16.5	1849.67
scene_5	baseline	2.33	200	16,318	10.0	2356.42
scene_5	proposed	2.31	220	8979	11.0	1927.60

**Table 5:** First-solution results in abstract mine-like scenarios.

Scene	Mode	Average Value				
		Time (s)	Expanded	Generated	RS Attempts	Cost
scene_1	baseline	42.45	4050	332,018	202.5	1364.89
scene_1	proposed	5.60	520	21,279	26	1156.33
scene_2	baseline	4.67	470	38,458	23.5	2528.85
scene_2	proposed	10.19	1260	51,619	63	2419.09
scene_3	baseline	1.95	160	13,038	8	1617.74
scene_3	proposed	4.04	400	16,359	20	1847.64
scene_4	baseline	1.55	130	10,578	6.5	1909.30
scene_4	proposed	1.04	80	3239	4	2065.22
scene_5	baseline	0.49	20	1558	1	2762.34
scene_5	proposed	0.46	20	779	1	3135.06

**Table 6:** Comparison of initial complete feasible-path planning performance among the baseline, IHA*, and proposed planners.

Scene	Case	Baseline			IHA*			Proposed		
		Success	Time (s)	Cost	Success	Time (s)	Cost	Success	Time (s)	Cost
scene_1	case_1	Yes	38.41	1451.23	No	29.56	–	Yes	4.13	1150.13
scene_1	case_2	Yes	46.50	1278.56	Yes	43.99	1795.86	Yes	7.07	1162.53
scene_2	case_1	Yes	5.90	2490.84	Yes	5.34	2775.76	Yes	7.07	2439.07
scene_2	case_2	Yes	3.45	2566.86	No	3.29	–	Yes	13.30	2399.11
scene_3	case_1	Yes	3.33	1207.62	Yes	66.65	1777.58	Yes	7.52	1079.86
scene_3	case_2	Yes	0.57	2027.85	No	3.45	–	Yes	0.56	2615.41
scene_4	case_1	Yes	0.70	2817.92	Yes	3.40	5184.19	Yes	1.06	2969.58
scene_4	case_2	Yes	2.40	1000.69	Yes	6.06	1548.78	Yes	1.01	1160.86
scene_5	case_1	Yes	0.50	3096.14	Yes	3.04	5507.71	Yes	0.46	3668.42
scene_5	case_2	Yes	0.48	2428.53	Yes	3.06	5610.34	Yes	0.46	2601.70

**Table 7:** Results in real-map-derived mining working-face scenarios with irregular obstacle boundaries.

Case	Method	Final Time (s)	Final Expanded	Final Generated	Final RS Attempts	Final Cost
case_1	baseline	687.25	25,680	2,105,678	1284	3395.98
case_1	proposed	515.29	23,900	1,314,445	1195	3171.76
case_2	baseline	10.69	380	31,078	19	1058.91
case_2	proposed	7.66	300	16,445	15	960.13
case_3	baseline	19.73	740	60,598	37	1496.20
case_3	proposed	13.05	560	30,745	28	1453.16
case_4	baseline	8.40	280	22,878	14	1595.04
case_4	proposed	7.67	280	15,345	14	1421.39

**Table 8:** Quantitative comparison in a real-map-derived mining working-face corridor scenario.

Metric	Baseline	Proposed	Improve
Final planning time (s)	17.015	14.022	17.59%
Final expanded nodes	940	900	4.26%
Final generated nodes	76,998	49,445	35.78%
Final RS attempts	47	45	4.26%
Final path cost	2627.80	1971.08	24.99%
First-solution time (s)	1.733	1.090	37.11%
First expanded nodes	80	60	25.00%
First generated nodes	6478	3245	49.91%
First RS attempts	4	3	25.00%
First-solution cost	3549.47	3931.33	−10.76%

## Data Availability

The data that support the findings of this study are available from the corresponding author upon reasonable request.
